# The evolution of surrogate microspheres in transarterial radioembolization: from SPECT prediction to multimodal AI-guided precision dosimetry

**DOI:** 10.7150/thno.134985

**Published:** 2026-08-12

**Authors:** Arun Gupta, Andrew C. Gordon, Samdeep K. Mouli, Robert J. Lewandowski, Dong-Hyun Kim

**Affiliations:** 1 Department of Radiology, Feinberg School of Medicine, Northwestern University, Chicago, IL 60611, USA.; 2 Department of Biomedical Engineering, McCormick School of Engineering, Evanston, IL 60208, USA.; 3 Robert H. Lurie Comprehensive Cancer Center, Chicago, IL 60611, USA.; 4 Department of Biomedical Engineering, University of Illinois at Chicago, Chicago, IL 60607, USA.

**Keywords:** transarterial radioembolization, surrogate, microspheres, hepatocellular carcinoma, radionuclides, Yttrium-90

## Abstract

Surrogate materials enable pre-treatment prediction of intra-hepatic distribution of therapeutic radioactive microsphere, facilitating patient-specific dosimetry for transarterial radioembolization (TARE). Technetium-99m macroaggregated albumin (⁹⁹ᵐTc-MAA) has long constituted the clinical standard for SPECT-based surrogates, however, its fundamental physicochemical difference from therapeutic microspheres limits the reliability of MAA-based dosimetry and prediction for personalized treatment planning. Next-generation surrogate technology includes biodegradable polymeric microspheres engineered to closely replicate the therapeutic microsphere while affording post-procedural arterial recanalization through controlled biodegradation. Theranostic microspheres incorporating diverse radionuclides, positron-emitting PET compatible surrogates, and multimodal CT and MRI-visible surrogates have collectively expanded same-particle pre-treatment dosimetry capabilities. These material-level advances are synergistically coupled with computational dosimetry tools including artificial intelligence/deep learning algorithms for automated segmentation/dose-prediction, and Monte Carlo simulations that enhance voxel-based dosimetry precision. This review examines recent advances in surrogate materials design and integrated imaging/dosimetry methodologies that enhance TARE efficacy, safety, and personalized treatment outcomes.

## 1. Introduction

Hepatocellular carcinoma (HCC), the most common primary liver cancer, is the third leading cause of cancer-related mortality worldwide 1. The majority of HCC cases about 70% are diagnosed at intermediate-to--advanced stages (BCLC stage B-D). Surgical resection and liver transplantation have been only curative interventions. However, eligibility is restricted to a minority of patients. Systemic tyrosine kinase inhibitors chemotherapy, immune checkpoint inhibitors immunotherapies, combinational chemo-immunotherapy regimes are also the therapeutic options but offer modest survival benefit. Consequently, local interventional therapies have been a primary disease management strategy across intermediate and advanced stages of HCC 2. Among local interventional therapies, transarterial radioembolization (TARE) has been proposed as a promising treatment modality for HCC treatment 3. The procedure involves hepatic intra-arterial infusion of radioactive microspheres, which exploit the selective tumor irradiation while minimizing damage to surrounding healthy tissues. The historical development of TARE spans more than seven decades. The earliest clinical applications employed radioactive gold in 1951, followed by the use of phosphorus-32 (^32^P) through the 1960s 4, 5. Yttrium-90 (^90^Y) subsequently emerged as the preferred and standard radionuclide for clinical TARE. As a pure beta emitter with a physical half-life of 64.1 hours and a mean soft-tissue penetration of approximately 2.5 mm (maximum ~11 mm), ^90^Y delivers effective locoregional tumor irradiation within a clinically manageable timeframe while limiting off-target dose deposition 6, 7. Although pure beta emitters like ^90^Y can be imaged using bremsstrahlung SPECT, the spatial resolution and quantification are limited due to its continuous energy spectrum. To address these imaging constraints, alternative radionuclides emitting both therapeutic beta and imageable gamma photons have been developed and investigated for TARE. Those radionuclides that have been investigated are summarized in **Table 1.**

Beyond radionuclide choices, the therapeutic efficacy of TARE is critically governed by the radionuclide carrier matrix or microsphere characteristics (size, material/composition, and morphology) as wells as by pre-procedural planning protocols including dosimetry. The size of microsphere delivering radionuclides for TARE is a principal determinant of lodgment depth within the tumor-feeding arteriolar bed, local radiation dose deposition, and the risk of non-target embolization. Oversized spheres occlude proximal feeding vessels and underdose viable tumor periphery, while undersized spheres risk traversing arteriovenous shunts to deposit in pulmonary or gastrointestinal vasculature 8, 9. Commercially available microspheres currently span a diameter range of 20 to 60 μm in diameter. However, the ideal size distribution for the best therapeutic efficacy remains under investigation. Matrix material composition is also regarded as critical consideration for their biocompatibility, cost-effectiveness, and structural integrity to withstand radiolytic stress.

**Figure 1:**
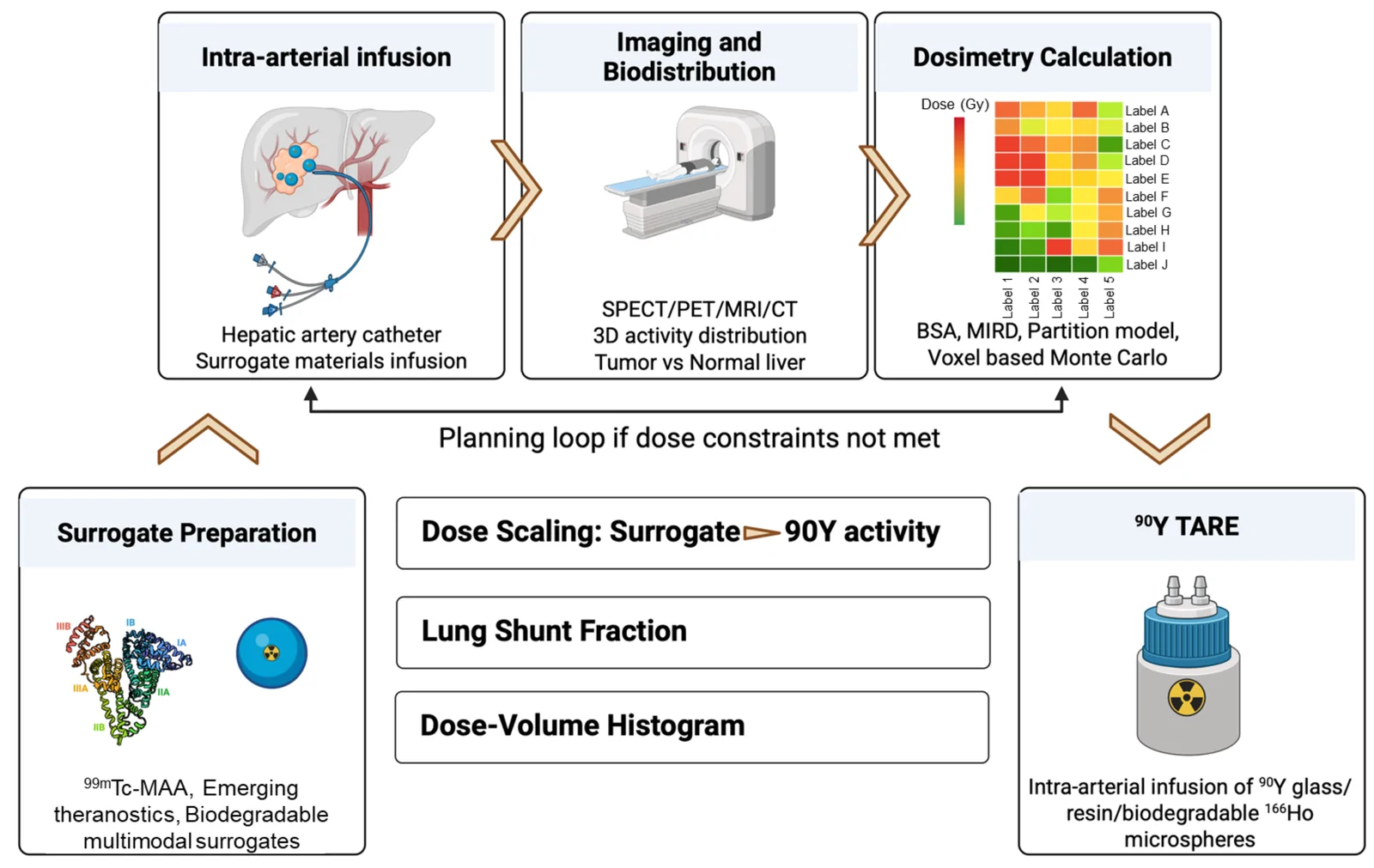
TARE workflow including dosimetry using surrogate materials and ^90^Y-microspheres.

A general stepwise overview of TARE procedure for HCC is illustrated in **Figure 1**. Pre-procedural planning protocols including dosimetry have one of the essential steps for successful TARE. ^99m^Tc-MAA has historically served as the standard surrogate for ^90^Y TARE pre-treatment dosimetry and planning. Recent advances have introduced several classes of next generation surrogate materials. Theranostic microspheres that integrate therapeutic radionuclide loading with intrinsic imaging capabilities offer promising options for precise treatment planning. Multimodal PET, MRI, and CT visible microspheres have also garnered significant attention with their enhanced spatial resolution and quantitative capabilities for three-dimensional patient-specific dose calculations 10. Biodegradable surrogate materials have further demonstrated favorable biocompatibility, reducing the risks of permanent off-target embolization 11. Complementing these material advances, AI-based dosimetry approaches are increasingly being integrated with novel surrogate platforms to enable highly personalized radiation delivery. This review systemically examines recent advancements in surrogate materials and pre-treatment dosimetry planning for TARE of HCC. We address the limitations of traditional surrogates and highlight emerging alternative surrogate materials. We further explore advanced voxel-based and AI-driven dosimetry strategies applicable in conjunction with these surrogate materials with the goal of achieving personalized dose prescription, therapeutic outcome optimization, and improved individual patient care in TARE of HCC.

## 2. FDA-Approved and CE-Marked Radioactive Microspheres for TARE Therapy

Currently, ^90^Y-glass microspheres (TheraSphere), ^90^Y-resin microspheres (SIR-Spheres), and ^166^Ho-poly-L-lactic acid (PLLA) microspheres (QuiremSpheres) have been developed for clinical TARE therapy in HCC. Their characteristics are summarized and compared in **Table 2**.

### 2.1 TheraSphere (^90^Y-glass microsphere)

TheraSphere, neutron-activated ^89^Y-dopped glass microspheres, was initially granted FDA Humanitarian Device Exemption (HDE) in 1999 and later obtained full Food and Drug Administration (FDA) approval in 2021 for the treatment of unresectable HCC, based on data from the LEGACY study 10, 12. This formulation shows high specific activity (approximately 2500 Bq/microsphere) and exceptional radionuclide stability. TheraSphere exhibits a narrow size distribution (20-30 μm), demonstrates minimal leaching (less than 0.13% after six weeks in saline) and has a relatively high density of 3.3 gcm^-3^ 10. While clinically established for HCC, the complex multi-step production processes yield only 10-20% of microspheres within the desired size range, affecting manufacturing inefficiency.

### 2.2 SIR-Spheres (^90^Y -resin microspheres)

SIR-Spheres are composed of resin microspheres with a broader size distribution (20–60 μm) and a density of 1.6 gcm^-3^ 10. FDA premarket approval (PMA) was issued in 2002 for the treatment of unresectable metastatic liver tumors 13. These ^90^Y-resin microspheres are produced by adsorbing ^90^Y onto their surface through an ion exchange process 10. The size distribution is relatively high, resulting in heterogeneous distribution in the liver and unpredictable vascular penetration depth, directly compromising dosimetric reproducibility. While SIR-Spheres offer simplified production methods, lower specific activity (approximately 50 Bq/microsphere) of the microspheres require a significantly higher number of microspheres to deliver a therapeutic dose.

### 2.3 QuiremSpheres (^166^Ho -PLLA microspheres)

QuiremSpheres are ^166^Ho-PLLA microspheres and only biodegradable option currently in clinical uses. These microspheres received CE mark approval in Europe in 2015 but remain under FDA consideration for clinical use in the United States 14. Manufacturing method is involving with the dissolving holmium acetylacetonate (^165^HoAcAc) and PLLA in chloroform, followed by neutron activation to generate ^166^Ho-PLLA microspheres. The size range of QuiremSpheres in diameter is from 15 to 60 μm and their density is the lowest (1.4 gcm^-3^) among commercial microspheres. The characteristic dual β and γ emissions of ^166^Ho and its paramagnetic properties enable multimodal SPECT and MRI imaging capabilities, facilitating enhanced precise treatment planning and distribution assessment 15. Nevertheless, there are several clinically and operationally relevant limitations. Comparatively shorter half-life (26.8 hours) and lower β energy than ^90^Y necessitate the administration of higher radionuclide activity quantities to achieve equivalent therapeutic doses, while narrowing the logistical schedule from the production to patient administration. The exclusive nuclear reactor dependency for the production introduces scheduling inflexibility. At the material level, the radiolytic PLLA degradation poses a structural integrity concern.

## 3. Clinical Surrogate Materials for TARE

While the therapeutic microsphere platforms have established the clinical foundation for TARE over two decades, the ultimate success of this approach depends critically on meticulous pre-treatment planning. The therapeutic efficacy of ^90^Y-TARE remains fundamentally constrained by the adequacy of intratumoral dose distribution, a parameter governed not only by operator-dependent angiographic mapping, but more critically, by the extent and spatial heterogeneity of tumor perfusion. Emerging dosimetric analyses from high-volume specialized centers have exposed a critical failure mode: incomplete ^90^Y dose coverage of the tumor volume is the primary driver of treatment failure, with 2-year local control rates falling below 10% in patients with hepatic metastases who receive suboptimal dosimetry 16, 17. Because dose deposition uniformity is inherently tied to microvascular perfusion architecture, reliable pre-treatment dosimetric prediction remains a major unsolved challenge. Here the global standard ⁹⁹ᵐTc-MAA and ⁹⁹ᵐTc-labeled alternative surrogates are summarized.

### 3.1. Clinical ^99m^Tc based Surrogates

#### 3.1.1.^ 99m^Tc-MAA Surrogates

**Figure 2:**
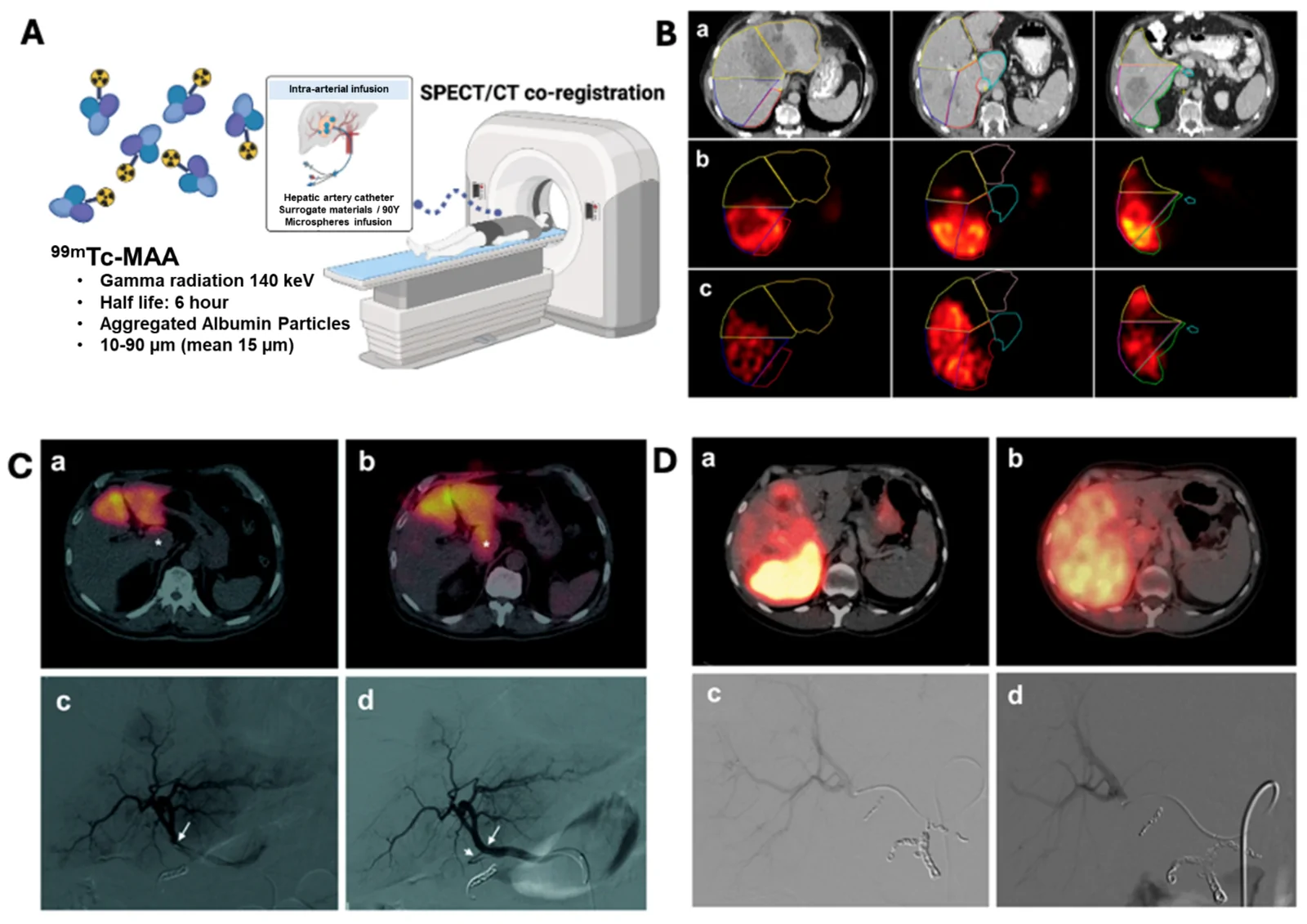
Intrahepatic distribution differences between ^99m^Tc-MAA and ^90^Y following hepatic artery administration 18. **(A)** ^99m^Tc-MAA imaging predicts the therapeutic distribution of ^90^Y microspheres. **(B)** Segmentations on contrast-enhanced CT images (top row, a) are co-registered with corresponding pre-treatment ^99m^Tc-MAA SPECT (middle row, b) and post-treatment ^90^Y SPECT (bottom row, c) images following activity injection into the right hepatic artery. Notable differences in intrahepatic activity distribution are observed between the ^99m^Tc-MAA and ^90^Y images within the same segmented regions. **(C)** Right-lobe radioembolization in a 36-year-old patient with colorectal liver metastases. Pre-treatment ^99m^Tc-MAA SPECT (a) and post-treatment ^90^Y SPECT (b) demonstrate marked discrepancies in microsphere distribution. Digital subtraction angiography (DSA) confirms identical catheter positioning in the right hepatic artery during both procedures (c, d), with coil embolization of the gastroduodenal, right gastric, and supraduodenal arteries. **(D)** Left-lobe treatment in a 72-year-old patient with uveal melanoma liver metastases. SPECT imaging shows significant differences between pre-treatment ^99m^Tc-MAA (a) and post-treatment ^90^Y (b) activity distributions, especially in the caudate lobe. DSA reveals variation in catheter tip positioning between injections: during ^99m^Tc-MAA administration (c), the catheter was placed distal to a key side branch, while during ^90^Y administration (d), it was positioned proximally, likely altering perfusion to the caudate lobe. Adapted with permission from 18, Copyright © 2013 Society of Nuclear Medicine and Molecular Imaging, Inc.

^99m^Tc-MAA is widely used as a standard surrogate material for pre-treatment imaging in ⁹⁰Y-TARE. This radiopharmaceutical facilitates dosimetry planning by simulating the anticipated biodistribution of therapeutic ⁹⁰Y microspheres, enabling assessment of tumor targeting efficiency, evaluation of hepatic perfusion patterns, and identification of potential non-target embolization risks. ^99m^Tc-MAA consists of aggregated albumin particles, with over 90% of the particles ranging between 10 and 90 µm in size (mean diameter: 15 µm), though some particles can reach 150 µm 18. The physical half-life is approximately 6 hours, and its gamma radiation at 140 keV is ideal for planner scintigraphy or SPECT imaging **(Figure 2A)**. Pre-treatment imaging with ^99m^Tc-MAA provides valuable insights into tumor targeting, extrahepatic deposition, and LSF estimation to prevent radiation-induced pneumonitis caused by hepatopulmonary shunting 19. ^99m^Tc-MAA’s ease of availability, cost-effectiveness, and compatibility with SPECT/CT imaging have established it as the standard surrogate material for ^90^Y-TARE.

**Figure 3:**
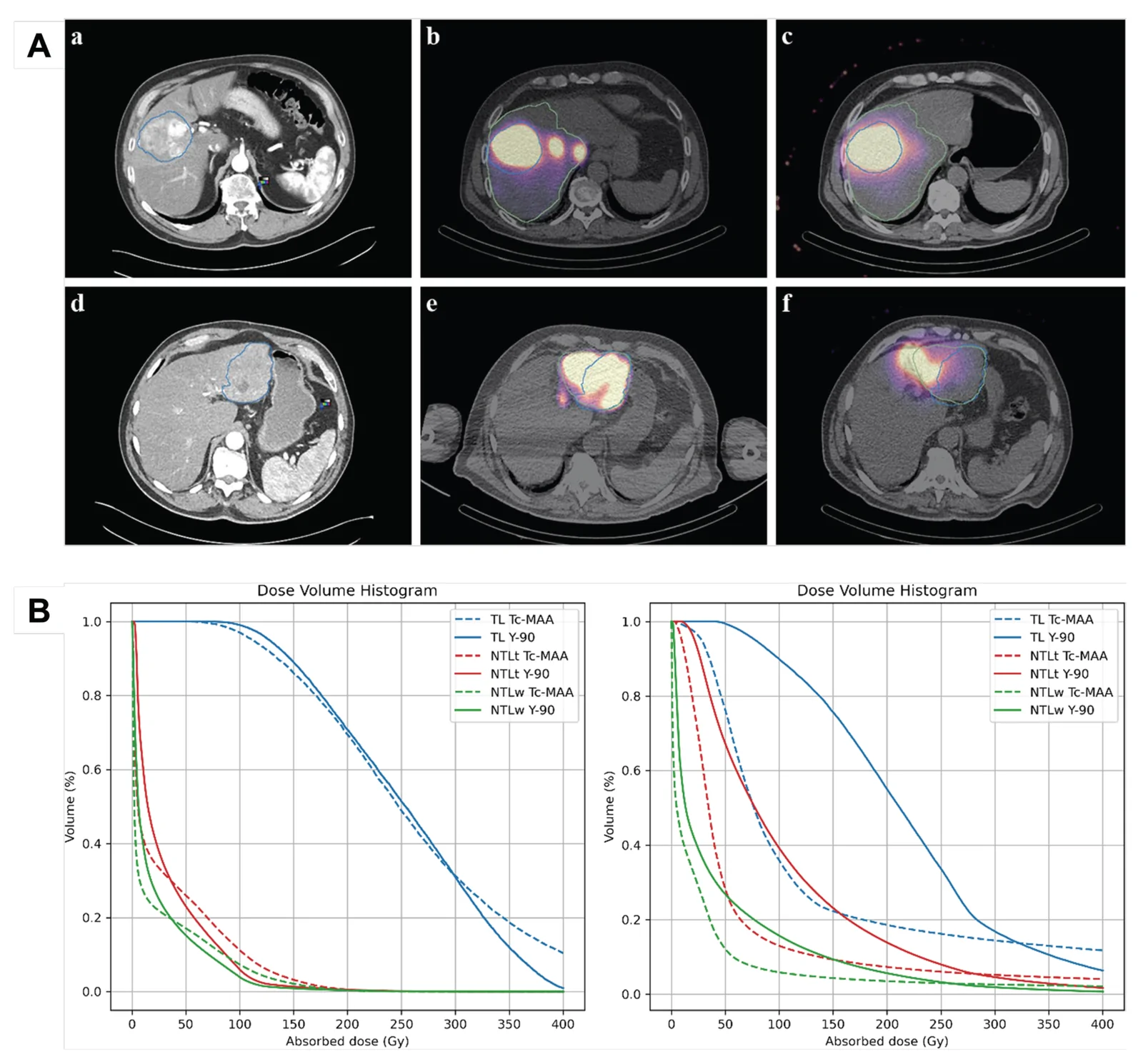
** (A)** Comparison of pre-therapy ^99m^Tc-MAA SPECT/CT and post-therapy ⁹⁰Y bSPECT/CT images. The top row (a-c) shows an example where simulation and therapy imaging exhibit good spatial agreement, while the bottom row (d-e) illustrates a case with clear mismatch between ^99m^Tc-MAA and ⁹⁰Y microsphere distributions. The tumor region (blue) and perfused liver lobe (green) are highlighted in both sets of images. **(B)** Dose–volume histograms (DVHs) for two HCC patients with a single lesion showing all VOIs (TL, NTLt, NTLw), illustrate a case with good visual agreement on SPECT (left) and a case with poor visual match (right). Adapted with permission from 21, Copyright © 2023 Springer Nature.

Despite its widespread use, ^99m^Tc-MAA exhibits notable discrepancies in biodistribution compared with ^90^Y microspheres, primarily due to the differences in particle size, density, vascular hemodynamics, and catheter positioning. These differences can result in suboptimal tumor targeting, unintended radiation to healthy tissues, and inaccurate estimations of extrahepatic shunting. Several studies have highlighted this mismatch; for example, Knesaurek *et al.* evaluated the reliability of ^99m^Tc-MAA SPECT/CT imaging quantitatively to predict the intrahepatic distribution of ^90^Y resin microspheres in 20 liver cancer patients 20. The authors concluded that exclusive reliance on ^99m^Tc-MAA imaging as a predictive surrogate for intra-hepatic distribution of ^90^Y microspheres can result in systemically discordant dosimetric estimates. Inconsistent and often suboptimal correlation between the two distributions has been observed across patients. Wondergem *et al.* further evaluated the predictive accuracy of ^99m^Tc-MAA SPECT for ^90^Y resin microspheres TARE 18. The results revealed more than 68% of liver segments exhibited discrepancies greater than 10%, as shown in **Figure 2**. In a related study, Riveira-Martin *et al.* reported a meaningful correlation between pre-therapy ^99m^Tc-MAA SPECT/CT and post-therapy ^90^Y bSPECT/CT dose metrices, supporting its potential role in TARE 21. However, ^99m^Tc-MAA consistently overestimated tumor absorbed dose by ~ 26% and showed mismatches in ^90^Y microspheres distribution **(Figure 3A)** and DVH analyses **(Figure 3B).** These discrepancies impacted dose prediction of tumoral and non-tumoral liver and were attributed to factors such as heterogenous tumor perfusion, necrotic regions, prior embolization, and arteriovenous shunting. These studies delineate the limitations of ^99m^Tc-MAA as a predictive tool and emphasize the need for more accurate imaging surrogates capable of individualized treatment planning. In response, alternative tracers including ⁹⁹ᵐTc-labeled human serum albumin (HSA) and sulfur colloid, have been investigated as surrogate candidates.

#### 3.1.2.^ 99m^Tc-labeled Alternative Surrogates

^99m^Tc-labeled HSA is an emerging alternative to ^99m^Tc-MAA, designed to produce particles between 20 and 50 µm that closely mimic the size and flow characteristics of ^90^Y microspheres 22. The structure of ^99m^Tc-labeled HSA is characterized by the coordination of a ^99m^Tc center to specific functional groups within the large, single-chain HSA protein. The improved stability and biocompatibility of HSA particles in physiological conditions compared to ^99m^Tc-MAA enhances their reliability during pre-treatment angiographic procedures and SPECT imaging. HSA-based tracers not only improve the accuracy of ^90^Y microsphere distribution predictions but also show potential in dual-tracer imaging. Grosser *et al.* compared the pharmacokinetics and liver–lung shunt (LLS) estimation of ^99m^Tc-MAA and ^99m^Tc-HSA in pre-treatment planning of TARE involving colorectal cancer patients and found similar liver and lung uptake at initial time points 23. However, while ^99m^Tc-MAA exhibited relatively rapid degradation over time, resulting in increased lung uptake and an overestimation of LLS at later imaging time points, ^99m^Tc-HSA demonstrated greater *in vivo* stability, with consistent biodistribution and minimal changes in LLS up to 24 hours post-injection. Authors concluded that ^99m^Tc-HSA is a more reliable and stable alternative compared to MAA for accurate LLS estimation in pre-treatment dosimetry. Furthermore, this approach augments the resolution of treatment planning and guides in detecting subtle shunting patterns that could be missed by ^99m^Tc-MAA alone. The limited commercial availability, non-standardized radiolabeling protocols, and inadequate validation of safety and efficacy have limited the widespread clinical adoption of ^99m^Tc-labeled HSA, particularly in anatomically challenging patients with complex vascular anatomy or significant arteriovenous shunting 24.

^99m^Tc-labeled sulfur colloid is another alternative to ^99m^Tc-MAA that has gained attention, especially for simultaneous dual-tracer hepatic-imaging strategy 25. Sulfur colloid is an inorganic colloidal dispersion synthesized through acid-precipitated heptasulfide which is structurally distinct from protein-based surrogates such as HSA or MAA. Technetium (^99m^Tc) is physically entrapped within a sulfur matrix, and the synthesis proceeds through the reaction of sodium thiosulfate (Na_2_S_2_O_3_) and a strong acid such as HCl in the presence of pertechnetate (^99m^TcO_4_^-^). The size of sulfur colloid particles ranges from 0.1 to 1.0 µm, smaller than ^99m^Tc-MAA particles. Colloidal particles undergo phagocytic clearance by liver, spleen, and bone marrow rapidly which provides complementary insights into vascular perfusion and embolization patterns 26. ^99m^Tc-labeled sulfur colloid is used to spatially differentiate perfusion patterns of tumor and non-tumor regions. It is especially utilized for complicated cases that showed marked intratumoral vascular heterogeneity arising from arterial neovascularization and extrahepatic shunting. Lam *et al.* performed ^99m^Tc-MAA and ^99m^Tc-sulfur colloid dual-tracer SPECT fusion imaging for physiology-based dosimetry in a patient with colorectal liver metastases 27. In this study,^ 99m^Tc-MAA simulated ^90^Y activity distribution whereas ^99m^Tc-sulfur colloid assessed liver function and perfusion. The absorbed doses to tumors (DT) and functional liver (DFL) were computed by fusing these images. DFL was correlated strongly with liver toxicity while DT was significantly associated with tumor response and survival. Patients receiving DT >55 Gy showed longer survival, highlighting the reliability of dual-tracer approach for personalized treatment planning. Sulfur colloids face several challenges such as translational constraints in production scalability and the requirement of specialized imaging protocols, which limit their routine clinical application.

### 3.2. Clinical Multifunctional Theranostic Surrogate Microspheres

The radioactive microspheres incorporating theranostic radionuclides, as mentioned in **Table 3,** demonstrate significant potential for the development of multifunctional TARE surrogate materials. Compared to the conventional workflows using two different radionuclides, these theranostics radioactive microspheres exploit both therapeutic beta emissions and gamma emissions for simultaneous imaging. This strategy facilitates precise tumor targeting, real-time dosimetry, and personalized treatment planning.

#### 3.2.1.^ 166^Ho-labeled microspheres

**Figure 4:**
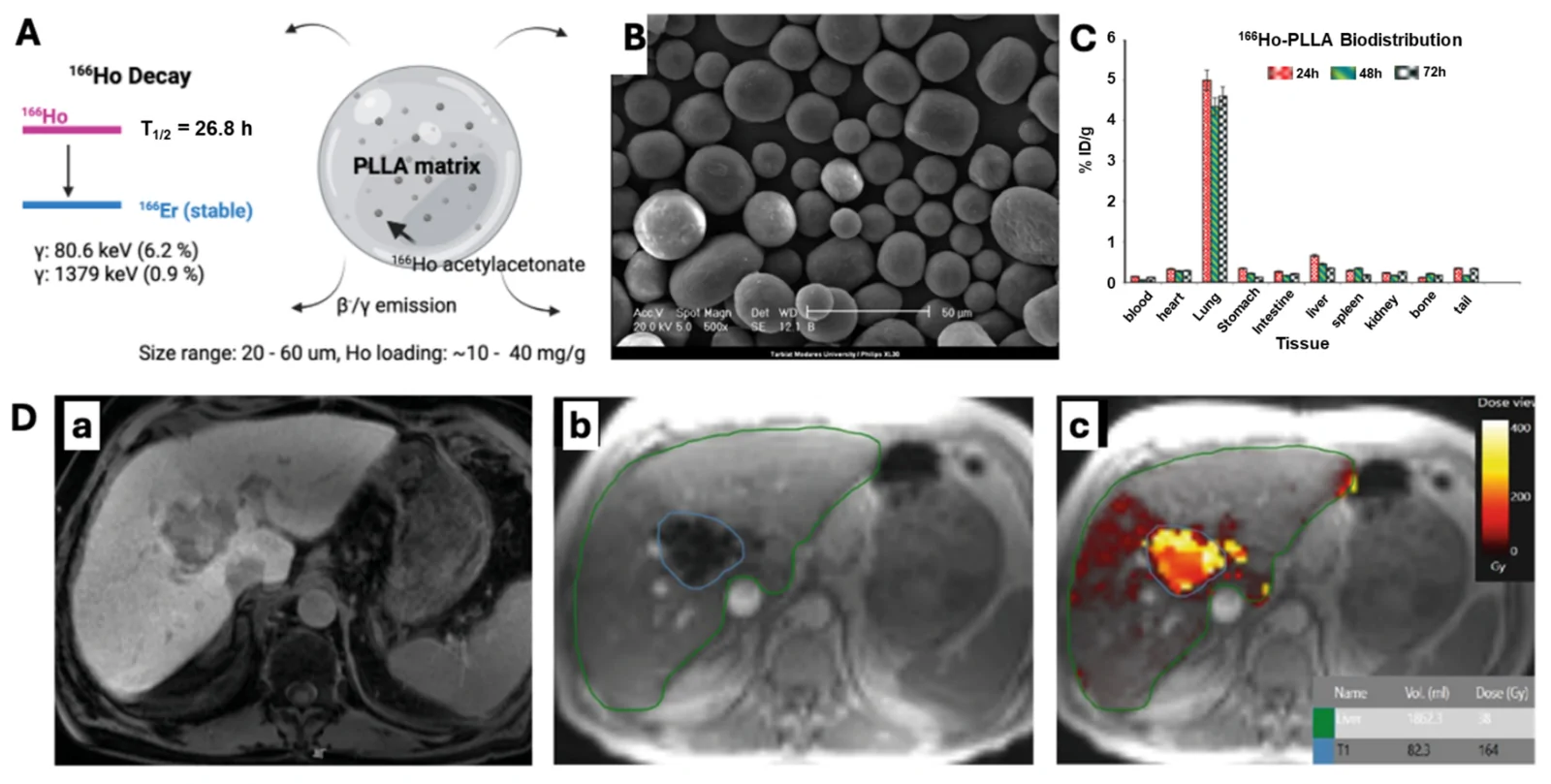
** (A)** ^166^Ho-PLLA microspheres. **(B)** Scanning electron microscopy images of ^166^Ho-PLLA microspheres after 1 hr neutron activation, showing preserved morphology post-activation. **(C)** Biodistribution of ^166^Ho-PLLA in wild-type male rats at 24, 48, and 72 hours following intravenous injection of 100 μCi, expressed as percentage of injected dose (%ID) (c). Each bar represents the mean ± standard deviation (n = 3). Adapted with permissions from 30, Copyright © 2015 John Wiley and Sons, Ltd.** (D)** Imaging of a 75-year-old male patient with HCC. Contrast-enhanced T1-weighted MR image showing a washout lesion in the right liver lobe (a). T1-weighted multi-gradient echo image post-^166^Ho radioembolization with liver (green) and tumor (blue) segmentations (b). Fused image combining the T1-weighted sequence with the calculated dose distribution (c); the grayscale bar represents absorbed dose in Grays. Adapted with permission from^33^, Copyright © 2019 Springer Nature.

^166^Ho-labeled microspheres represent advanced theranostic agents, combining therapeutic beta emissions with gamma rays (81 keV, 6.7%) suitable for SPECT imaging, enabling simultaneous treatment delivery and real-time dosimetry assessment 31, 33. ^166^Ho-loaded microspheres, first introduced for TARE by Nijsen *et al*., have since been validated through multiple preclinical studies demonstrating their safety, low toxicity, and therapeutic efficacy 65. The paramagnetic properties of ^166^Ho allow for MRI-based 3D mapping of microsphere distribution, significantly enhancing treatment planning through MRI-guided dosimetry 15, 29. Typically, ^166^Ho is incorporated into **poly(L-lactic acid) (PLLA)** microspheres, with a size range of **20–60 µm (Figure 4A) 29, 30, 33.** Yavari *et al.* reported the synthesis, characterization, and dosimetry evaluation of ^166^Ho-PLLA microspheres for potential use in TARE therapy 30. The microspheres are produced by a solvent evaporation of ^165^Ho-acetylacetonate, followed by neutron activation, and show high *in vitro* stability and radiochemical purity (>99%). The study demonstrated excellent particle integrity and surface characteristics even after neutron irradiation, as shown in** Figure 4B.** Dosimetry and biodistribution data in rats confirmed high lung retention (~85% at 72 h), minimal off-target activity, and excellent *in vivo* stability. Subramanian *et al.* developed ^166^Ho-labeled **Biorex™ 70 resin microsphere** as a cost-effective, indigenously manufactured alternative to commercially available TARE platforms, demonstrating a favorable mean particle size (~68 μm) 31. Excellent hepatic retention (~95%) with minimal off-target accumulation was demonstrated in rats. Clinical validation in the HEPAR trials have further demonstrated strong dose-response relationship and the safety and efficacy of ^166^Ho microspheres for personalized dosimetry in TARE therapy 32, 36. Additionally, the scout dose of **^166^Ho-PLLA microspheres (QuiremScout™)** in pre-treatment imaging ensured accurate dose delivery with minimized discrepancies commonly observed in ^99m^Tc-MAA surrogates 33, 34. However, the lower specific activity of ^166^Ho microspheres compared to other commercially available microsphere devices may require higher treatment volumes, underscoring the need of additional clinical data to optimize the safety and efficacy 32, 66.

#### 3.2.2.^ 188^Re-labeled microspheres

^188^Re has been extensively investigated as a theranostic radionuclide for TARE 39, 40, 42, 44, 45. The suitable gamma emission (155 keV, 15%) of ^188^Re facilitates precise SPECT imaging for accurate dosimetry and enhanced therapeutic planning 41. A variety of carrier materials such as lipiodol and biodegradable matrices such as HSA, poly-lactic acid (PLA), starch, and PLLA have been used to formulate ^188^Re-microspheres with the size ranges from 10 to 100 µm, suitable for effective tumor targeting and selective biodistribution 39-42, 44, 45. In several preclinical studies and clinical trials, excellent radiolabeling efficiency, *in vitro* stability, and selective hepatic tumor targeting have been reported with ^188^Re-microspheres. Delaunay *et al.* used ^188^Re-Lipiodol in the Phase 1 Lip-Re I clinical trial and demonstrated favorable biodistribution and selective liver retention (>90%), high tumor uptake, and excellent tumor-to-normal tissue dose ratios. They highlighted the potential of ^188^Re-Lipiodol as a theranostic agent for TARE in HCC 43. Similarly, Vega *et al.* fabricated uniform sized ^188^Re-PLA microspheres (~40 µm) and demonstrated selective tumor localization, high tumor-to-normal tissue dose ratios, and compatibility with SPECT imaging in HCC rat models. These promising theranostic microspheres need further studies to optimize microsphere formulations for uniform size and validate their efficacy through large-scale clinical trials.

#### 3.2.3.^ 153^Sm-labeled microspheres

The dual functionality of ^153^Sm facilitates real-time SPECT imaging via its gamma emission (103 keV, 28% abundance), allowing accurate mapping of microsphere distribution and improving the precision of dose calculation in TARE therapy 46, 47, 49, 51. The neutron activation of ^152^Sm provides a scalable and cost-effective production pathway, making ^153^Sm-microspheres particularly accessible in regions with limited radiopharmaceutical infrastructure. Several microparticles, such as Amberlite resin, polystyrene, polymethacrylate, and others, have been utilized to develop ^153^Sm-microsphere formulations 46, 49, 51. However, polymers like acrylic, PLLA, and polyhydroxybutyrate-co-3-hydroxyvalerate (PHBV) offer enhanced biocompatibility and significantly reduce long-term embolic risks through their biodegradability 47, 48, 50. The typical size range of these microspheres is presented in **Table 3**. In a study, Wong *et al.* developed ^153^Sm-acrylic microspheres (~35 µm) and demonstrated high radionuclide retention and excellent labeling efficiency, making them a safer and biocompatible alternative to ^90^Y-microspheres for TARE 47. Alregib *et al.* reported the production of ^153^Sm-PHBV microspheres (~30 µm) co-loaded with doxorubicin for chemo-radioembolization. These ^153^Sm-microspheres demonstrated high radionuclide retention and dual cytotoxic effects for the treatment of advanced HCC 50. The activation of ^152^Sm to ^153^Sm requires nuclear reactors, limiting the widespread adoption of ^153^Sm microspheres for clinical TARE therapy.

#### 3.2.4.^ 131^I-labeled microspheres

**Figure 5:**
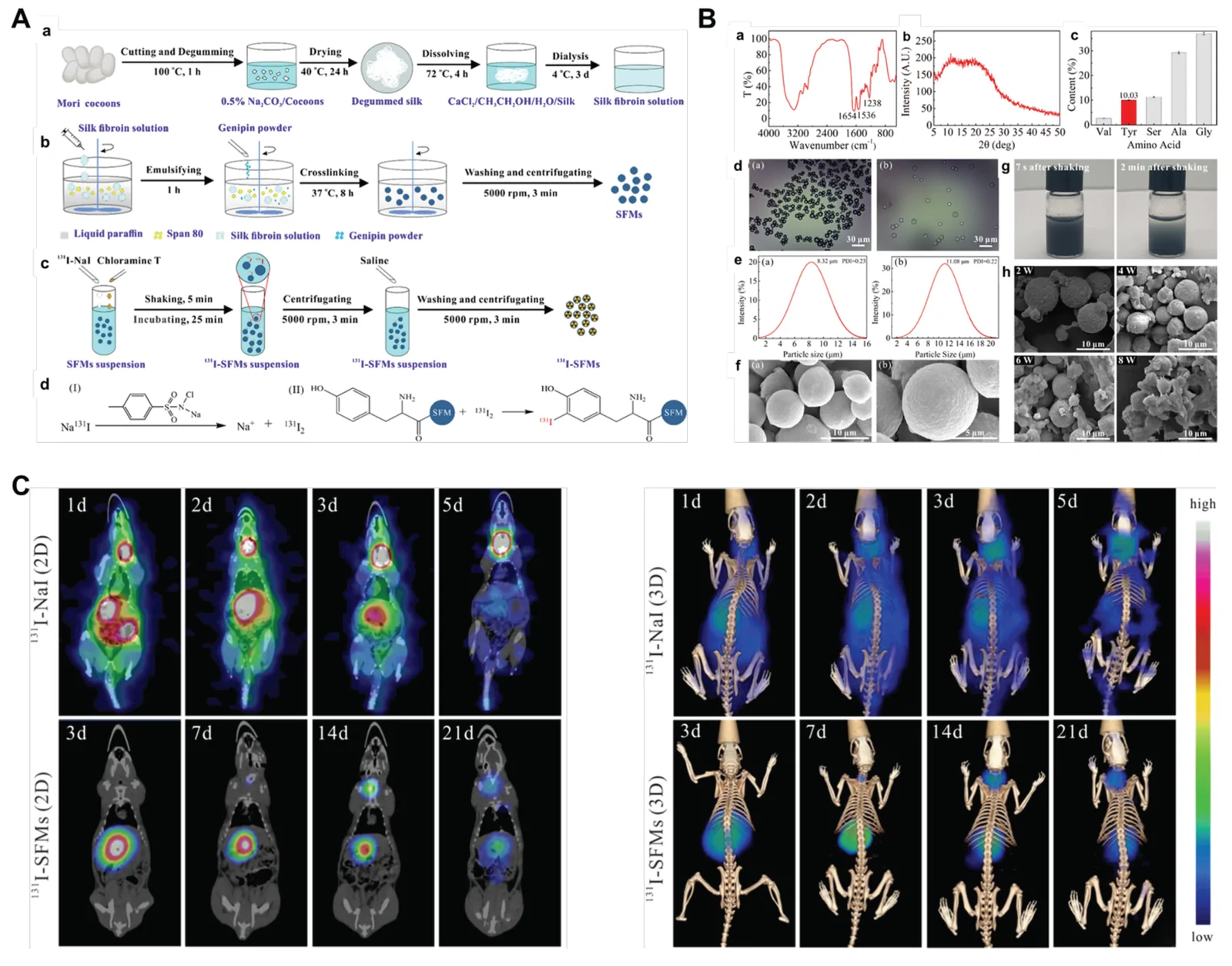
Development, characterization, and *in vivo* imaging of ^131^I-labeled silk fibroin microspheres (SFMs) for TARE in a rat HCC model. **(A)** Schematic workflow of the synthesis and labeling process: extraction of silk fibroin from Bombyx *mori cocoons* (a); preparation of SFMs via emulsification and genipin crosslinking (b); radiolabeling of SFMs using the chloramine-T method to yield ^131^I-SFMs (c); chemical reaction mechanism of iodination on tyrosine residues (d). **(B)** Physicochemical and morphological characterization: FTIR (a) and XRD spectra (b) of extracted silk fibroin confirming secondary structure; amino acid composition revealing 10.03% tyrosine content (c); optical microscopy of air-dried and swollen SFMs (d); particle size distribution showing ~11 μm diameter (e); SEM confirming spherical morphology (f); suspension stability of SFMs (g); SEM images over 8 weeks showing biodegradation in rat serum (h). **(C)** In vivo SPECT/CT imaging comparing ^131^I-NaI and ^131^I-SFMs biodistribution post transarterial administration. ^131^I-NaI shows rapid systemic spread and thyroid uptake (2D and 3D views). In contrast, ^131^I-SFMs demonstrate persistent hepatic retention and localized signal up to 21 days, confirming stable embolization and targeted delivery to liver tumors. Adapted with permission from 57, Copyright © 2022 American Chemical Society.

^131^I-labeled Lipiodol gained significant acceptance in the past for the treatment of HCC, particularly in patients with portal vein thrombosis due to their selective tumor accumulation 54, 67, 68. The favorable gamma emissions profile (364 keV, 81%) of ^131^I further supports developing novel theranostic microspheres using different biodegradable materials, such as chitosan, silk fibroin, and chitosan-collagen composites 53, 55-57. The size range of ^131^I-labeled biodegradable microspheres is presented in **Table 3**. Several preclinical studies have evaluated the potential of these microspheres for selective tumor embolization, enhanced biodistribution, and natural degradation to minimize long-term embolic risks. For instance, Hwang *et al.* demonstrated that ^131^I-labeled chitosan hydrogels (100–120 µm) effectively targeted hepatic tumors and acheived significant tumor growth suppression and minimal extrahepatic deposition in rodent models 55. In another study, Wu *et al.* synthesized ^131^I-labeled silk fibroin microspheres (SFM) around 11 µm diameters with optimal radiolabeling efficiency and demonstrated excellent biodistribution, biocompatibility, and tumor inhibitory effects in rat models 57. **Figure 5** illustrates the stepwise synthesis process, characterization, and *in vivo* preclinical SPECT/CT imaging performance of SFM and the confirmation of their effective embolization and targeted radiation delivery in rat HCC. Challenges such as dosimetry optimization, off-target effects, and large-scale validation remain to be addressed to enhance their clinical utility.

#### 3.2.5.^ 177^Lu-labeled microspheres

**Figure 6:**
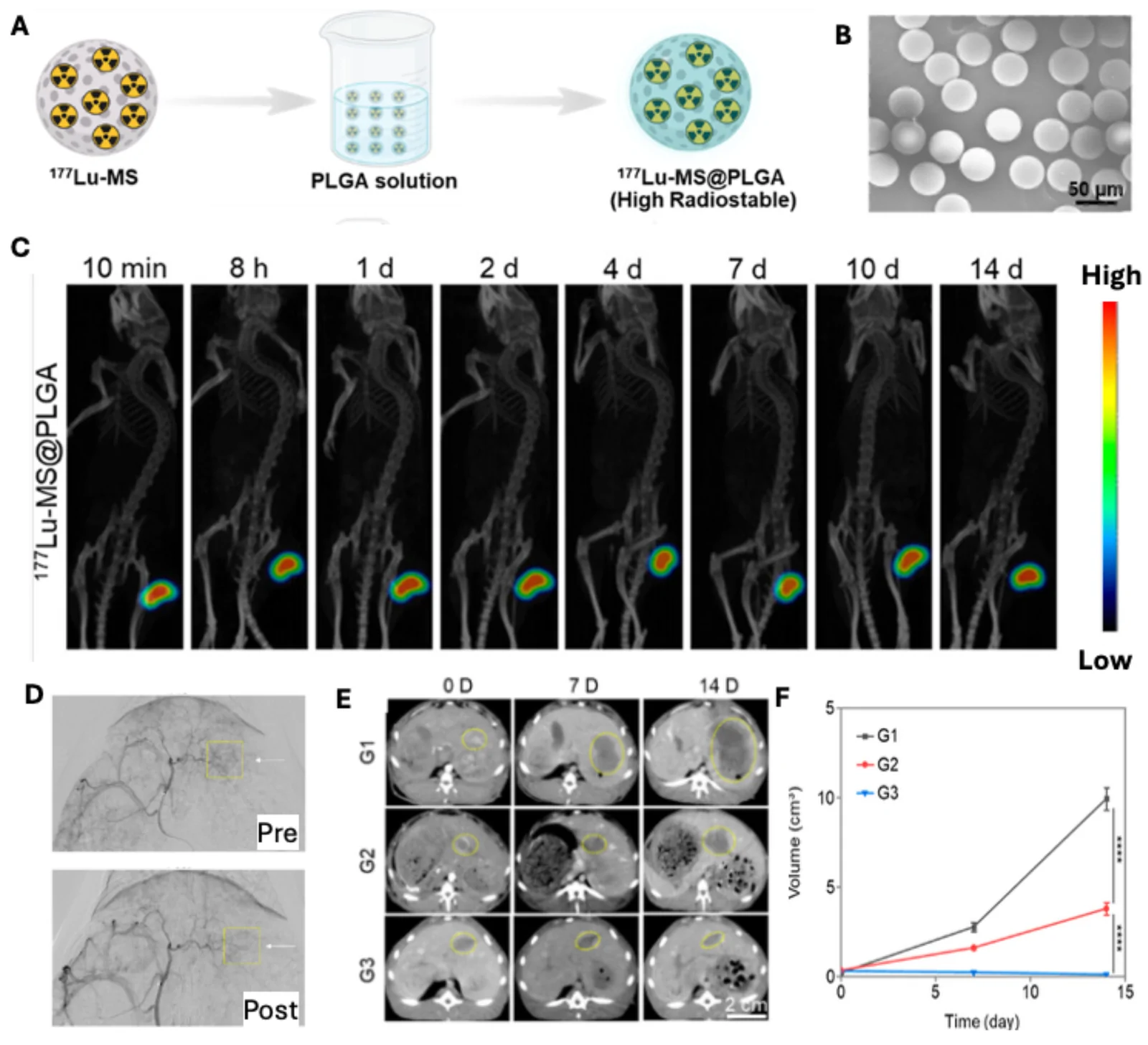
** (A)** Fabrication of PLGA-coated ^177^Lu-labeled hollow mesoporous silica microspheres (^177^Lu-MS@PLGA) using PLGA coating method. **(B)** Scanning electron microscopy image of PLGA-coated ^176^Lu-MS@PLGA. **(C)** MicroSPECT/CT images of HepG2 tumor-bearing mice demonstrate intratumoral retention of ^177^Lu-MS@PLGA. **(D)** Digital subtraction angiography (DSA) images obtained before and after intra-arterial embolization highlights the embolization site (yellow box). **(E)** Serial enhanced CT scans of VX2 liver tumors in three treatment groups; G1: PBS (control), G2: non-radioactive MS@PLGA, and G3: ^177^Lu-MS@PLGA reveal substantial tumor growth suppression in G3. **(F)** Tumor volume quantification over 14 days shows significantly greater inhibition in the ^177^Lu-MS@PLGA group compared to controls. Adapted with permission from 61, Copyright © 2024 American Chemical Society.

^177^Lu-labeled radiotracers have been successfully utilized in the treatment of neuroendocrine tumors and prostate cancers due to their low energetic β-particles (497 keV) and optimal γ-emission (113 and 208 keV) for SPECT imaging-based precise dosimetry and treatment planning 62, 69, 70. Leveraging this success, various surrogate materials such as chitosan, PLGA, polydopamine (PDA), and alginate are now being investigated for the development of ^177^Lu-labeled microspheres for preclinical TARE applications 58-62. These ^177^Lu-labeled microspheres are often engineered to a range of 20–60 µm, ensuring optimal embolization and selective delivery within tumor vasculature, thus minimizing the risk of leakage and non-target embolization. Several preclinical studies have demonstrated their efficacy and safety in HCC models. For instance, Jin *et al.* developed ^177^Lu-labeled PLGA-coated silica microspheres (^177^Lu-MS@PLGA) by applying a simple PLGA coating over ^177^Lu-labeled hollow mesoporous silica particles, which demonstrated high radiolabeling efficiency, exceptional *in vitro* and *in vivo* radiochemical stability and minimal off-target leakage **(Figure 6)** 61. ^177^Lu-SPECT/CT of rabbit further demonstrated sustained tumor retention, low systemic distribution, and significant tumor growth inhibition. Recently, Wu *et al.* produced multifunctional PDA-coated ^177^Lu-microspheres that provide both TARE and photothermal therapy. The ^177^Lu-SPECT imaging showed precise microspheres localization and enhanced therapeutic effects 59. The results from these studies collectively highlight the potential of ^177^Lu-labeled microspheres as a safe, imageable, and highly effective theranostic platform for TARE. The high production cost and limited availability of ^177^Lu-labeled microspheres currently constrain its broad clinical adoption. Additional optimization in microsphere fabrication and large-scale clinical validation is crucial to firmly establishing its clinical application.

#### 3.2.6.^ 142^Pr-labeled microspheres

^142^Pr has shown great potential for theranostic TARE applications due to its beta particles (2.162 MeV) and gamma photons (1.575 MeV, 3.7%,) emissions 64, 71. ^142^Pr-labeled microspheres are produced through neutron activation of ^141^Pr embedded within rare-earth aluminosilicate glass 64. The favorable density of ^142^Pr-labeled microspheres (~4 g/cm³) enables successful embolization and effective radiation delivery 64, 71. ^142^Pr-microspheres offer higher biological effective doses (BEDs), particularly for aggressive tumors when compared to ^90^Y. The dose distributions are comparable, and the therapeutic delivery is faster due to their shorter half-life (19.12 hours) 64. Further preclinical research and clinical validation are essential in expanding the efficacy and accessibility of ^142^Pr-microspheres for TARE therapies.

#### 3.2.7. ¹⁷⁵Yb-labeled microspheres

Previously applied for bone pain palliation and rheumatoid arthritis, ^175^Yb-labeled microspheres have been recently proposed for TARE as a promising and innovative approach to treat unresectable HCC 72-74. The favorable half-life (4.2 days) and excellent decay characteristics such as β-particles (470 keV) and γ-emissions (133 keV, 282 keV, and 396 keV) of ^175^Yb are well suited for simultaneous imaging and effective therapy 72. In a study, Jamre *et al.* demonstrated the fabrication and radiolabeling of biodegradable ^175^Yb-PLLA microspheres (20–40 μm) via neutron activation where they obtained high radiochemical purity and exceptional specific activity 63. Preclinical studies in mice displayed complete tumoral retention of radioactivity for 48 hours, subsequently suppressed tumor growth from day 4 post-injection and showed significant tumor necrosis at day 12 post-injection. Imaging data showed significant tumor-specific uptake with low systemic distribution, highlighting their potential for intra-tumoral radiotherapy of HCC 63. Further dosimetry studies and clinical investigations of ¹⁷⁵Yb microspheres can lead to a new treatment option for TARE therapy.

#### 3.2.8. Scout-dose resin (scout^90^Y) and ^166^Ho-PLLA (QuiremScout™) microspheres

**Figure 7:**
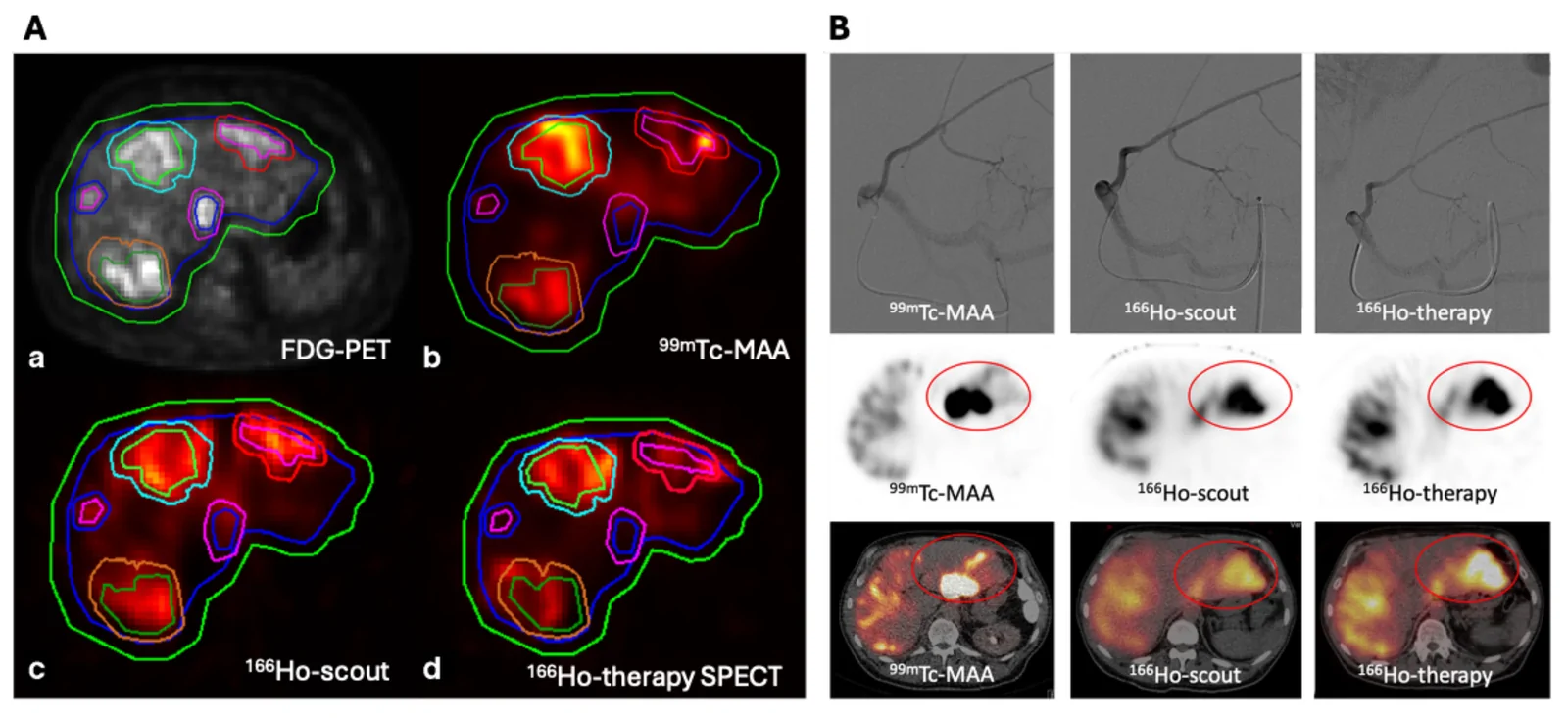
Comparison of predicted and delivered microsphere distributions using conventional (^99m^Tc-MAA) and bioidentical surrogates (^166^Ho-scout).** (A)** Segmentation of liver and tumor volumes overlaid on FDG-PET and co-registered SPECT images acquired with ^99m^Tc-MAA, ^166^Ho-scout, and post-therapy ^166^Ho. The additional 1 cm margin illustrates dose coverage regions for comparison across modalities. **(B)** Representative case demonstrating a clear mismatch between ^99m^Tc-MAA and the ^166^Ho-therapeutic dose distribution. Despite identical catheter positioning (top row), SPECT-CT images (middle and bottom rows) show that ^166^Ho-scout closely mimics the therapeutic distribution, while ^99m^Tc-MAA deviates substantially. Adapted with permission from 36, 2020 Springer Nature.

The concept of scout-dose administration using bioidentical microspheres, such as resin-based ^90^Y (scout^90^Y) and ^166^Ho-PLLA (QuiremScout™), has emerged as a more accurate alternative to traditional surrogates like ^99m^Tc-MAA for predicting therapeutic microsphere distribution 34, 36, 75. Unlike ^99m^Tc-MAA, these scout agents share identical physical and embolic properties with their therapeutic counterparts. Kokabi *et al.*, demonstrated that resin-based ^90^Y scout doses (mean administered activity of 560 MBq) provides strong correlation with therapeutic biodistribution, particularly in non-segmental treatments where ^99m^Tc-MAA is known to be less reliable 75. Similarly, ^166^Ho scout microspheres allowed dual-modality imaging (SPECT and MRI) and providing enhanced visualization and precision dosimetry. Radosa *et al.*, demonstrated that a pre-therapy scout dose (~250 MBq) accurately predicted therapeutic distribution by MRI-based real-time visualization and personalized dose optimization prior to treatment **(Figure 4D)** 33. In a similar study, Smits *et al.* reported strong correlation between ^166^Ho-PLLA scout and therapeutic dose distributions compared to ^99m^Tc-MAA with narrower 95% limits of agreement for lesion absorbed dose (-90.3 to 105.3 Gy vs. -164.1 to 197.0 Gy) 36. As illustrated in **Figure 7,** ^166^Ho-scout closely matched with the distribution of therapeutic microspheres (^166^Ho-Therapy), whereas ^99m^Tc-MAA showed a clear discrepancy in SPECT-CT images. Clinically, this improved predictive accuracy with ^166^Ho-scout has critical implications for the treatment planning.

## 4. Emerging Surrogate Microspheres

### 4.1. Biodegradable surrogate microspheres

**Figure 8:**
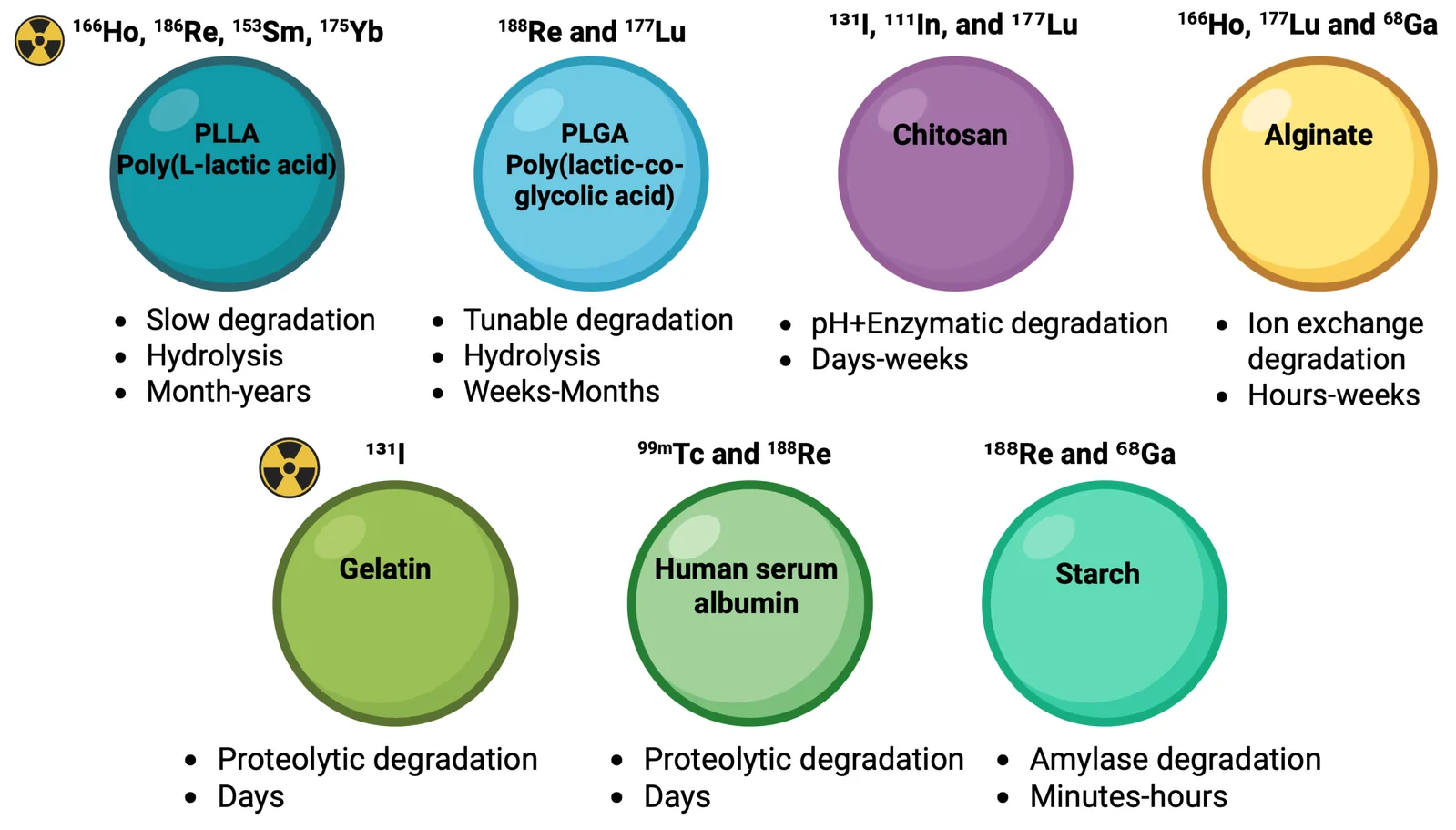
** Degradation mechanisms of biodegradable surrogate microspheres for radioembolization and drug delivery.** Schematics of representative biodegradable polymers that can be used for surrogate microspheres and their dominant degradation mechanisms. Synthetic PLLA and PLGA undergo bulk hydrolytic ester bond cleavage with autocatalytic acceleration due to acidic byproducts. The tunable degradation is over weeks to months depending on composition and crystallinity. Polysaccharides (chitosan, alginate, starch) are primarily degraded via enzymatic glycosidic bond cleavage or ionic crosslink dissociation. Chitosan exhibits lysozyme- and pH-responsive degradation, alginate undergoes Ca²⁺ crosslink exchange and network destabilization, and starch is rapidly cleaved by α-amylase, resulting in transient embolic behavior. Protein-based materials (gelatin, human serum albumin) degrade predominantly through proteolytic enzymatic pathways, with kinetics modulated by crosslink density and local inflammatory milieu. Together, these degradation mechanisms govern *in vivo* residence time, embolic stability, and clearance kinetics, which are critical parameters for designing next-generation biodegradable surrogate microspheres in TARE.

Non-degradable surrogate microspheres pose limitations for repeated TARE procedures due to their permanent embolic effect and potential to obstruct future treatment. While ⁹⁹ᵐTc-MAA is the current clinical standard surrogate for both resin and glass microsphere-based TARE, it exhibits limited correlation with actual microsphere distribution. To overcome these challenges, biodegradable surrogate microspheres have been explored as alternative surrogates for pre-treatment planning 76-78. Ideally, these microspheres should exhibit biocompatibility, biodegradability, and physiochemical properties including size, shape, and density-comparable to the therapeutic microspheres, to better replicate their intrahepatic distribution. The transient embolic effect of biodegradable microspheres facilitates vascular restoration, thereby enabling repeated treatment cycles without impairing future TARE procedures 79. Additionally, their degradation into non-toxic byproducts minimizes the risk of ectopic embolization. Several natural and synthetic polymers have been employed to develop such biodegradable microspheres for both preclinical and clinical TARE applications **(Figure 8)** 22, 56, 62, 80.

#### 4.1.1. Poly(L-lactic acid) Microspheres

Poly(L-lactic acid) (PLLA) **is a biocompatible and** biodegradable polymer extensively used for the synthesis of biodegradable microspheres labeled with various radionuclides, including ^166^Ho, ^186^Re, ^153^Sm and ^175^Yb for TARE applications 31, 33, 44, 63, 65. **PLLA** degrades into **non-toxic lactic acid byproducts** via hydrolysis, ensuring safe clearance from the body, reducing **long-term embolic risks and systemic toxicity**, and enhancing its safety profile as a promising alternative to non-degradable microspheres 29. Once lodged in the capillaries, polymer chains in PLLA microspheres slowly break down into smaller polyester fragments absorbed and recycled as lactic acid, a process taking up to 3 to 6 months at 37°C. Initial spherical morphology is maintained through the therapeutic window for less than 5 days. Progressive hydrolytic fragmentation reduces intact sphere diameter over weeks, with complete fragmentation into submicron lactic acid oligomers by 3~6 months, restoring capillary patency 81, 82. Several preclinical and clinical studies have shown that **PLLA microspheres** labeled with ^166^Ho effectively accumulate in liver tumors with minimal deposition in non-target organs 30, 31, 33, 35. **Yavari *et al.*** validated the ***in vivo* stability of** ^166^Ho-PLLA **microspheres in Wistar rats**, demonstrating their **radioactive purity and effective tumor targeting**. Jamre *et al.* prepared biodegradable PLLA microspheres loaded with ^188^Re sulfide colloidal nanoparticles (^188^Re-SC-PLLA) demonstrating excellent *in vitro* stability and sustained localization in mouse models, highlighting their potential as effective agents for radioembolization of liver tumors 44.

#### 4.1.2. Poly(lactic-co-glycolic acid) Microspheres

Poly(lactic-co-glycolic acid)** (**PLGA) is a well-established synthetic biodegradable polymer used extensively in drug delivery and medical implants due to its **FDA-approved safety profile** and ability to degrade into non-toxic byproducts (lactic acid and glycolic acid), which are naturally metabolized by the body 83-85. PLGA is an ideal candidate for fabricating radioactive microspheres due to its versatility, biocompatibility, and tunable degradation rates. Several preclinical and clinical studies have been performed that exhibit the potential of radiolabeled PLGA microspheres for tumor-specific radioembolization 61, 85. For example, **Shukla *et al.* sythesized^188^Re-DMSA loaded in PLGA microspheres,** achieving **high radiolabeling efficiency (>97%)** and radiochemical **stability 85. Recently,** Jin *et al*. produced ^177^Lu-labeled PLGA-coated microspheres (^177^Lu-MS@PLGA) with a mean diameter of ~42.6 μm. These PLGA coated microspheres showed enhanced radiochemical stability due to minimal leakage of ^177^Lu in biological environments, thus **improving tumor retention (Figure 6)** 61. Furthermore, the PLGA allows the microspheres to degrade into non-toxic byproducts (CO₂ and water), offering excellent biosafety for radioembolization therapy. The favorable characteristics of PLGA microspheres such as size, morphology, degradation kinetics and the ability to encapsulate and release radionuclides in a controlled manner makes them superior and promising biodegradable surrogate for effective radioembolization therapy.

#### 4.1.3. Chitosan Microspheres

Chitosan is a positively charged natural polysaccharide derived from the deacetylated chitin 58. Excellent biocompatibility, biodegradability, and low toxicity have been demonstrated. Especially, abundant amine and hydroxyl functional groups of chitosan facilitate various radiolabeling with radioisotopes such as ¹³¹I, ¹¹¹In, and ¹⁷⁷Lu. Those radioactive chitosan microspheres (Chi-MS) have been proposed for a promising alternative microsphere TARE platform of HCC 55, 56, 58, 62. **¹³¹I-labeled chitosan microspheres** exhibited effective tumor targeting, prolonged hepatic retention, and significant tumor growth inhibition in preclinical rat models 55. Additionally, **¹³¹I-labeled chitosan-collagen microspheres** developed by Pang *et al.,* showed **high radiolabeling efficiency along with enhanced biodegradability and suspension stability 56.** Novel approaches incorporating **¹⁷⁷Lu-labeled chitosan microspheres 58** and ¹⁷⁷**Lu-polydopamine-coated Chi-MS with magnesium oxide nanoparticles 62** have also been explored. Favorable radiochemical stability, embolization effectiveness, and hypoxia mitigation were well verified. However, the relatively **slow degradation rate of chitosan-based microspheres** is **less suitable for transient embolization of surrogates 62.** The **linear polymer structure and solubility constraints in acidic media also** pose additional challenges for clinical applications 58.

#### 4.1.4. Gelatin Microspheres

Gelatin is a fibrous protein derived from the partial hydrolysis of collagen. Its biodegradability, biocompatibility, non-toxicity, hydrophilicity, and non-immunogenicity has been well-proved, and it is widely regarded as a safe material for a broad range of biomedical applications 86, 87. Given gelatin’s safety feature, gelatin-based microspheres represent a promising platform, and have been explored as a carrier for radionuclides used in TARE of HCC 52, 53. Ma *et al.* reported ¹³¹I-labeled gelatin microspheres (¹³¹I-GMSs) designed for TARE. Hepatic artery infused ¹³¹I-GMSs was visualized with SPECT imaging. Localized ¹³¹I-GMSs in the liver with minimal thyroid accumulation and following degradation over 32-48 days were observed, suggesting slow iodine release and *in vivo* safety 52. Another preclinical study by Chi* et al.* demonstrated high therapeutic efficacy of ¹³¹I-GMSs in a nude mouse HCC model, with extended survival rates relative to control (73.3% vs. 13.3%, P < 0.001). SPECT imaging confirmed selective tumor localization of ¹³¹I-GMSs with minimal off-target accumulation while microspheres remained in tumor tissue for up to 32 days 53. Additionally, the degradation rate of gelatin can be controlled through the degree of crosslinking, indicating its strong potential as a degradable surrogates platform for TARE of HCC 88.

#### 4.1.5. Alginate Microspheres

**Figure 9:**
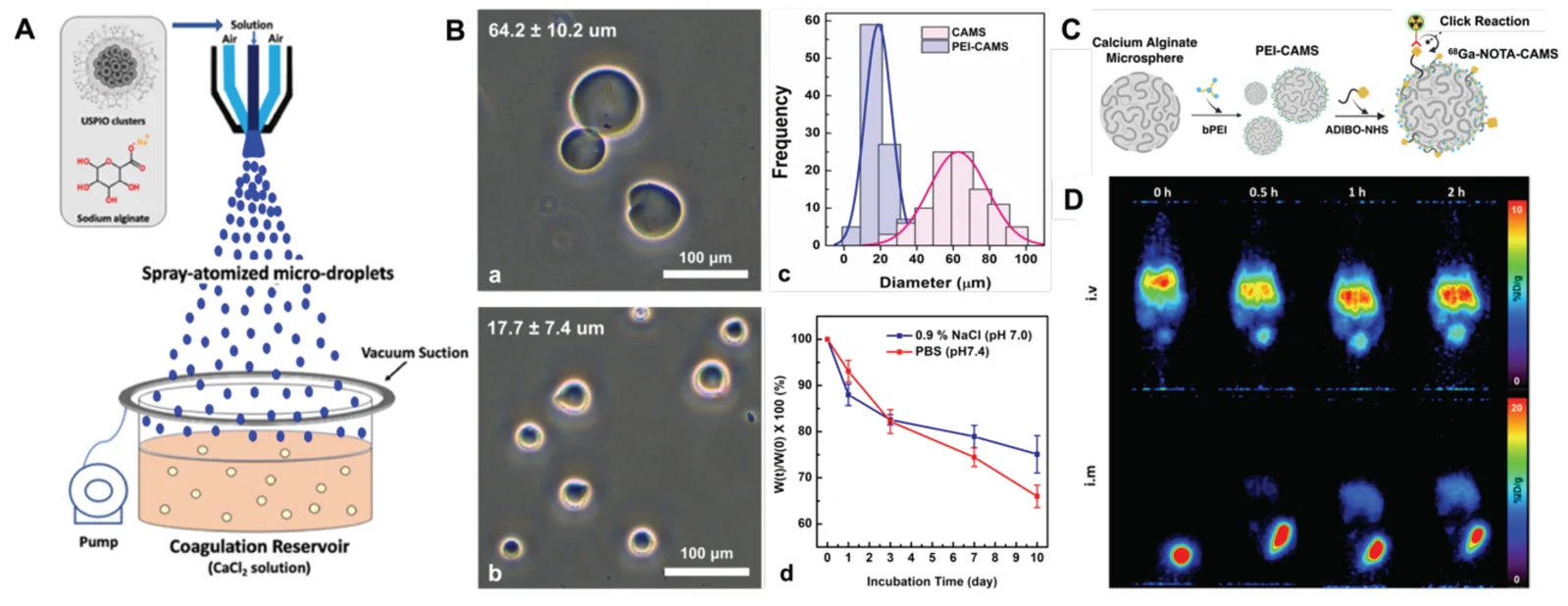
** (A)** Schematic showing calcium alginate microspheres fabrication process using spray-coagulation method. Adapted with permission from 95, Copyright © 2020 Elsevier Ltd. **(B)** Optical images of CAMSs (a, 64.2 ± 10.2 μm) and PEI-CAMSs (b, 17.7 ± 7.4 μm); (c) shows corresponding particle size distributions, and (d) illustrates in vitro degradation profiles in 0.9% NaCl and PBS over 10 days, demonstrating up to ~35% mass loss in PBS. **(C)** Schematic showing surface modification of PEI-CAMSs with ADIBO-NHS followed by radiolabeling with [⁶⁸Ga]Ga-NOTA-N₃ via click chemistry reaction. **(D)** Preclinical PET images acquired at 0–2 h post-injection showing stable localization of [⁶⁸Ga]Ga-NOTA-PEI-CAMSs at the injection site after intravenous (i.v.) and intramuscular (i.m.) administration in mice, confirming *in vivo* stability. Adapted with permission from 89, Copyright © 2024 American Chemical Society.

Alginate is a polysaccharide derived from brown algae. Its biocompatibility, biodegradability, hydrophilicity, and non-toxicity have been well validated, and widely adopted for a range of biomedical applications 89. It can form hydrogels upon exposure to divalent or trivalent cations such as calcium or lanthanides, making it an ideal matrix for embolization therapy 87. Recent advancements have further enhanced their functionality by incorporating radioisotopes such as ^166^Ho, ^177^Lu and ^68^Ga for the potential application in radioembolization therapy. Zielhuis *et al.* developed^ 166^Ho-loaded alginate microspheres and demonstrated their multimodal imaging capability via MRI and gamma imaging 28. The intra-arterial injection into the renal artery of a pig successfully embolized the left kidney, demonstrating the feasibility of TARE therapy. Yang *et al.* developed ^177^Lu-labeled alginate microspheres for radio-immunotherapy in HCC using mouse and rabbit models. In murine model, intratumoral injection of 2 µm microspheres with anti-PD-L1 immunotherapy inhibited primary tumor growth and suppressed distant tumors by modulating the tumor immune microenvironment. In rabbits, intra-arterial injection of 40 µm microspheres successfully embolized the central auricular artery, demonstrating the potential of these microspheres for TARE therapy 60. Recently, Gupta *et al.* fabricated polyethyleneimine (PEI) decorated calcium alginate microspheres and labeled with ^68^Ga (^68^Ga-PEI-CAMS) as PET imaging surrogates for potential application in TARE 89. The microspheres fabricated via a spraying–coagulation method exhibited a uniform spherical shape with an average size of ~17.7 ± 7.4 μm after PEI coating **(Figure 9)**. The *in vitro* degradation studies confirmed 25–35% mass loss within 10 days under physiological conditions, supporting their biodegradability and safety profile. Favorable radiolabeling efficiency and *in vivo* stability allowed PET imaging of the microspheres. The promise of alginate-based biodegradable microspheres is suggested as a safer and versatile alternative to non-degradable surrogates for pre-treatment imaging and accurate dosimetry in TARE.

#### 4.1.6. Human Serum Albumin (HSA)

HSA microspheres are one of clinical translatable biodegradable platform with favorable biocompatibility and minimal immunogenicity. Inherent versatile binding sites enable stable radionuclides conjugation 22, 40. Additionally, their resistance to radiolysis makes them a potentially stable radioactive microsphere. These microspheres conjugated with radionuclides such as ^99m^Tc and ^188^Re have been extensively studied for a surrogate material of TARE 23, 39. Wunderlich *et al.* demonstrated that ^188^Re-HSA microspheres exhibit high stability *in vivo* while maintaining tumor-selective accumulation and minimizing systemic exposure 39. The biodegradable matrix of HSA microspheres enables complete resorption within weeks to months, reducing the long-term risk of embolic complications of ischemia and inflammation compared to non-degradable microspheres. Alrfooh *et al.* highlighted the biodegradability of HSA microspheres as a safer alternative to permanent microspheres 22. However, radiolabeling efficiency and radiolabeling stability* in vivo* is relatively low, limiting their widespread clinical adoption. To address these limitations, various synthesis methods, including oil emulsion and microwave-assisted techniques, have been developed to optimize HSA microsphere production 22.

#### 4.1.7. Starch-based Microspheres (SBMP)

SBMPs are biodegradable embolic agents with crosslinked start polymers that can undergo enzymatic degradation *in vivo*. Their established safety combined with cost effectiveness has positioned SBMPs as a promising translatable platform for transarterial embolization 22. Degradable starch microspheres (DSM), such as EmboCept® S DSM 35/50, are commercially available and primarily used for transient intra-arterial hepatic chemoembolization, reducing post-embolization syndromes. The degradation can occur primarily via enzymatic hydrolysis by serum alpha-amylase within 35-40 minutes 90. Various SBMPs stooges have explored their labeling with co-radionuclides such as ¹⁸⁸Re and ⁶⁸Ga for theranostic applications 41. Radiolabeled SBMPs are generally synthesized by oxidizing starch with sodium periodate, followed by the functionalization with polyamine ligand (e.g., cadaverine) for radiometal chelation. Verger *et al.* reported high-efficient radiolabeled ¹⁸⁸Re- and ⁶⁸Ga-SBMPs using ready-to-use radiolabeling kits. Developed radiolabeled ⁶⁸Ga-SBMPs were delivered into hepatic artery in HCC rat models, and their preferential tumor accumulation was successfully monitored by PET/CT imaging 41. Future clinical studies are critical in validating the dosimetry, safety, and therapeutic efficacy of radiolabeled SBMPs theranostic surrogate platform for image-guided TARE.

### 4.3. Positron Emitting Surrogate Microspheres

SPECT-based surrogate microspheres have several limitations, including production challenges, inconsistent particle sizes, and non-availability of radioactive microspheres with high-specific-activity. In addition, pre-treatment SPECT imaging with these microspheres suffers from lower spatial resolution compared to PET imaging, limiting the precise dose calculations and detailed biodistribution mapping **(Table 4)**. PET surrogate microspheres, on the other hand offers superior imaging capabilities and effective therapy planning through PET image guided MC dosimetry.

Several positron-emitting radionuclides, including fluorine-18 (^18^F), gallium-68 (^68^Ga), copper-64 (^64^Cu), yttrium-86 (^86^Y), and zirconium-89 (^89^Zr), have been exploited to develop PET-compatible surrogates for ^90^Y microspheres 41, 89, 91-94. Selwyn *et al.* fabricated ^18^F-labeled resin microspheres (size range: 20–40 µm) as PET surrogates for ^90^Y resin microspheres 92. These microspheres were efficiently radiolabeled and showed high *in vitro* stability. *In vivo* rabbit study also demonstrated PET monitoring of favorable ^18^F-labeled resin microspheres biodistribution. PET imaging provided superior resolution and quantification for accurate dosimetry for treatment planning. Similarly, Avila-Rodriguez *et al.* developed ^86^Y-, ^89^Zr-, and ^64^Cu-labeled resin microspheres sized with 20–40 µm 91. Efficient radiolabeling and excellent *in vitro* stability were demonstrated. PET imaging of the radiolabeled resin microspheres in rats showed sufficient* in vivo* stability for ^86^Y and ^89^Zr microspheres, while ^64^Cu showed significant leaching. Demonstrated superior resolution and quantification of PET imaging capability with the radiolabeled resin microspheres will permit precise tumor-to-normal tissue ratio assessments and enhanced dosimetry. However, the non-degradable nature of resin microspheres poses challenges with long-term biocompatibility and potential embolic risks. In another approach, Larson *et al.* fabricated ^68^Ga-infused silica microspheres using a microfluidic technique. They generated stable and nearly monodisperse (10–50 µm) microspheres with excellent *in vitro* stability without significant leaching 94. However, their study was limited due to the lack of *in vivo* validation and microspheres biodegradability data.

To address the shortcomings of traditional microspheres designs, later studies focused on novel materials and methods to fabricate PET microspheres. ^68^Ga- and ^188^Re-labeled SBMP with a mean particle size of 29.65 ± 11.73 µm were developed for both pre-treatment PET imaging and TARE therapy 41. The *in vivo* studies of ^68^Ga-SBMP showed successful intra-arterial administration in a DENA-induced HCC rat model, with >95% localization of ^68^Ga-SBMP in the liver, particularly in tumors. ^68^Ga-PET/CT confirmed minimal off-target distribution and no systemic recirculation, demonstrating the stability and potential of ^68^Ga-SBMP as a PET-compatible microspheres for pre-treatment imaging and personalized dosimetry. Moreover, the biodegradability of SBMP, ease of generator-based ^68^Ga production and improved spatial resolution and quantitative accuracy of PET imaging position them as promising alternatives to existing surrogate microspheres. In another study, Chambers *et al.* introduced ^18^F-labeled ceramic hydroxyapatite microspheres of 20–30 µm with high radiochemical stability 93. The PET imaging in rats, following intravenous tail vein injection, demonstrated selective hepatic accumulation and biodistribution consistent with ^90^Y-microspheres. Recently, ^68^Ga-labeled alginate-based biodegradable PET microspheres developed by Gupta *et al.* demonstrated excellent properties as a promising surrogate for preoperative imaging in TARE therapy 89. The biodegradability of alginate, combined with its straightforward click chemistry-based radiolabeling, resulted in excellent radiolabeling efficiency (>99%) and high in vitro radiochemical stability (>92%) in both PBS and human serum. Preclinical PET imaging **(Figure 9)**, further confirmed *in vivo* stability and biodistribution, supporting their potential application as PET surrogates. However, PET-compatible microspheres have several limitations, including the short half-life of radioisotopes, radionuclide leakage, generation of inconsistent particle size, material degradability, and limited preclinical and clinical data. PET-compatible microspheres and their characteristics have been summarized in **Table 5.** Additional research should focus on designing PET microspheres with optimal particle size to accurately mimic the behavior of ^90^Y microspheres. Further, robust preclinical and clinical studies using the transarterial infusion approach are needed to establish their role as imaging surrogates for ^90^Y in HCC treatment. In addition to these technical limitations, the clinical translation of PET-based surrogate microspheres is influenced by cost and feasibility considerations. Compared to the widely utilized ^99m^Tc-MAA, which is relatively inexpensive, PET radionuclides such as ^18^F and ^68^Ga require more advanced and expensive production methods, imaging infrastructures and radiochemistry support, increasing overall procedural costs. While generator-produced ^68^Ga improves accessibility relative to cyclotron-dependent tracers, their implementation remains limited in resource-constraints settings. PET-based surrogates offer superior spatial resolution and quantitative accuracy; however, their routine clinical application may be restricted to specialized centers or complex cases where high-precision dosimetry is critical. Therefore, PET-compatible microspheres are currently best considered as complementary tools to ^99m^Tc-MAA, with their adoption guided by clinical need, resource availability, and the anticipated benefit in personalized dosimetry.

### 4.3. Radiopaque Microspheres

Transmission imaging modalities, such as CT and digital subtraction angiography, are widely used for their high resolution and diagnostic accuracy, often enhanced by radiopaque contrast agents that improve visualization of anatomical structures 96. This principle has been extended to the development of radiopaque microspheres by incorporating high atomic number materials, such as iodine, tantalum, gold or barium sulfate, into polymeric microspheres 97, 98. For example, iodine-modified acrylamido-polyvinyl alcohol (PVA-AMPS) microspheres (70-300 μm), such as LC Bead LUMI™ and DC Bead LUMI™ demonstrated excellent radiopacity during embolization procedures and are FDA-approved for use in hypervascular tumors and arteriovenous malformations 99, 100. Zeng *et al.* investigated tantalum nanoparticle-loaded alginate microspheres (Ta@CaAlg), which exhibited radiopacity comparable to Iodixanol, a commercial contrast agent 101. Renal artery embolization in rabbits demonstrated their long-term visibility and efficacy, highlighting their potential for TARE applications. Radiopaque microspheres provide real-time visualization, facilitating precise targeting of hepatic tumors in TARE. Moreover, their imageability allows interventional radiologists to modify the treatment strategy during the TARE procedure using CT imaging, improving the chances of achieving adequate absorbed doses across all tumor regions.

Recently, Eye90 microspheres, developed by ABK Biomedical, have emerged as innovative radiopaque microspheres for embolic therapies for TARE 8, 102-104. Eye90 microspheres are composed of a proprietary radiopaque glass composition containing high atomic number compounds, designed to closely resemble glass (TheraSphere) microspheres in size (20–30 μm diameter) and density (3.4 g/cm³). The ^90^Y in Eye90 microspheres is generated through thermal neutron absorption of ^89^Y embedded within the glass matrix. The radiopacity of Eye90 microspheres enables real-time localization of microspheres using high resolution CT imaging, primarily addressing the limitations of limited spatial resolution associated with ^90^Y PET and bremsstrahlung SPECT 103. Henry *et al.* conducted several phantom studies to establish and validate a quantitative relationship between CT Hounsfield Units and radiopaque microsphere concentration and showed the potential of CT-based dosimetry in ^90^Y TARE 102, 103. This group demonstrated superior dose distribution visualization, reduced partial volume effects, and enhanced representation of dose heterogeneity in preclinical rabbit liver models using CT-based dosimetry. In a first-in-human clinical trial, Abraham *et al.* assessed the safety, therapeutic effectiveness, and imaging capabilities of radiopaque Eye90 microspheres in individuals with unresectable hepatocellular carcinoma, using a dual-syringe system for intraarterial delivery 8. The results revealed strong agreement between microsphere localization seen on CT and radioactivity patterns observed on SPECT/CT, enabling real-time visualization of tumor targeting. The inherent radiopacity of Eye90 microspheres allowed the detection of heterogeneous dose distributions and highlighted areas of insufficient tumor coverage. The treatment was well-tolerated, and most of the patients exhibited either complete or partial responses at both 90- and 180-day post-treatment. Overall, the study underscored the potential of CT-visible microspheres to enhance precision in ^90^Y radioembolization and enable future CT-based dosimetry.

### 4.4. MRI-visible Microspheres

MRI-visible microspheres enable high-resolution imaging of microspheres distribution and precise dose measurement without ionizing radiation 105-107. MRI-visible microspheres are classified into T1-weighted agents (e.g., gadolinium-based compounds) and T2-weighted agents (e.g., superparamagnetic iron oxide nanoparticles; SPIONs). Li *et al.* fabricated SPIO-labeled yttrium microspheres ranging from 20 to 40 µm in diameter, composed of yttria-alumina-silicate (YAS) glass with 2-20% SPIO. They demonstrated that microspheres with 2% SPIO content exhibit a strong correlation between MR imaging R2* and microsphere concentrations (R² = 1.00, P < .001) 108. In animal models, their study reported that MR-based dose quantification showed high accuracy, with a strong correlation between R2 values and the infused microspheres dose (ICC = 0.98, P < .001). In a study, Qin *et al.* designed MRI-visible polymer microspheres by embedding magnetic ferrite nanoclusters (FNs) into a poly(acrylic acid) hydrogel matrix 109. These microspheres fabricated using a microwave-assisted solvothermal reduction method were ranged in size from 100 to 900 µm. Their study demonstrated that T₂-weighted MRI signals decreased proportionally with increasing microsphere concentration, reflecting strong susceptibility effects. The *in vivo* experiments confirmed that subcutaneously injected magnetic microspheres in mice remained clearly visible on MR images for up to 28 days. Some researchers have developed polyvinyl alcohol (PVA) hybrid microspheres incorporating gadolinium oxide (Gd₂O₃) and iron oxide (Fe₃O₄) to enable dual T1/T2 MRI imaging 110. Another approach includes iron oxide-loaded tris-acryl microspheres and holmium-lipiodol-alginate microspheres, which allow visualization across multiple imaging modalities, including MRI and CT 111, 112. However, the microspheres described above, such as polymer-based and PVA hybrid formulations incorporating Gd₂O₃ and Fe₃O₄ remain in preclinical stages and have not yet been utilized for TARE due to size limitations or incomplete validation of biocompatibility. In contrast, ^166^Ho-PLLA microspheres have been extensively studied for TARE. Their inherent MR visibility allows for mapping of microspheres distribution and dosimetry via quantitative MRI and SPECT. Various clinical trials, including the HEPAR studies have demonstrated that ^166^Ho-microspheres enable accurate assessment of intrahepatic microspheres deposition and allow for integrated imaging-therapy workflows using single agent 14, 15, 29, 32, 33, 36, 65. Among MRI-visible microspheres, both SPIO-labeled microspheres and ^166^Ho-PLLA microspheres holds strong promise for MRI-guided treatment planning and post-treatment dosimetry in TARE.

### 4.5. ^99m^Tc-radiolabeled Nanoparticles Composites

**Figure 10:**
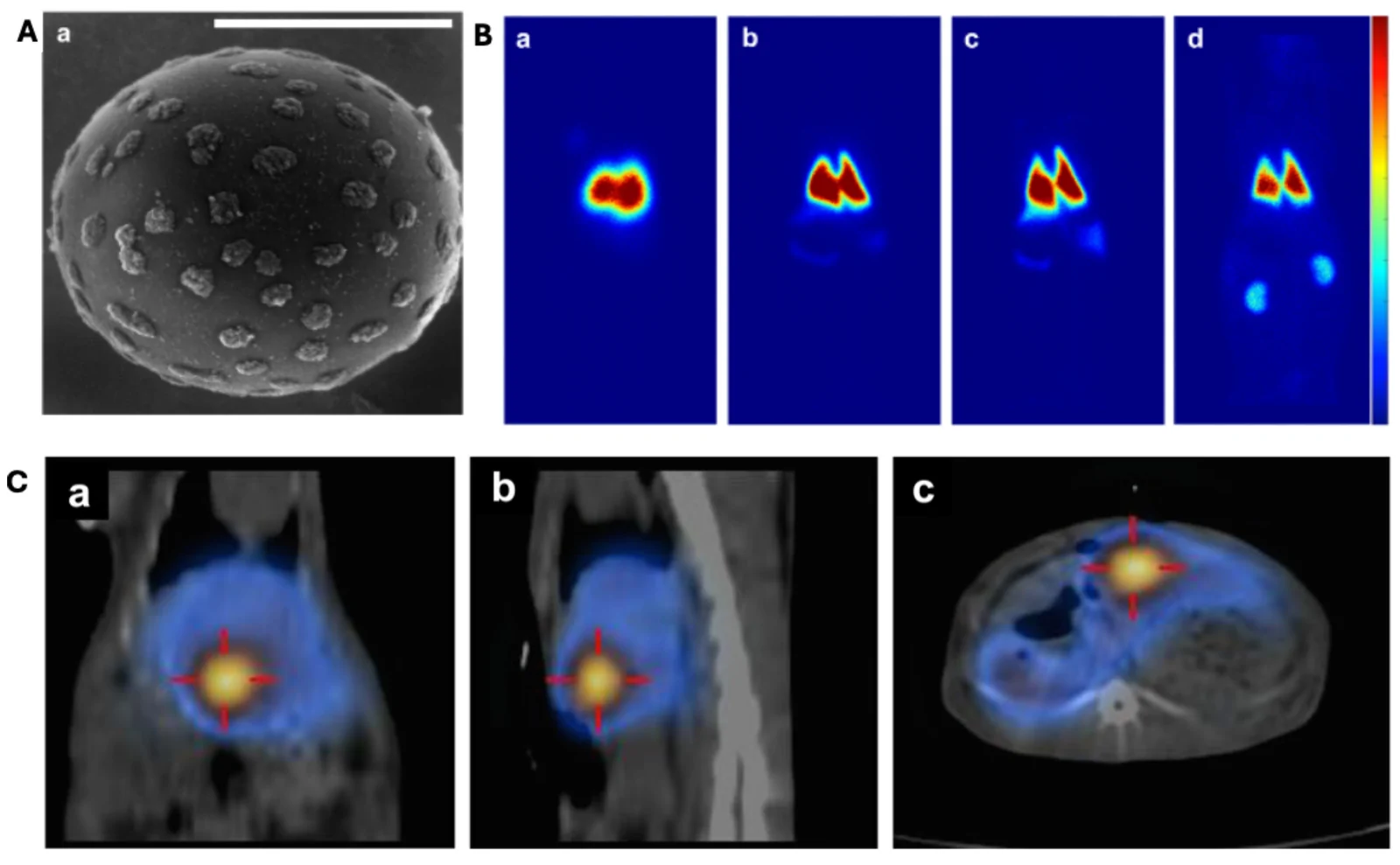
Characterization and imaging performance of ^99m^Tc-labeled nanoparticle composite microspheres 113. **(A)** Scanning electron microscopy (SEM) images showing the surface morphology of radiolabeled microspheres with protamine-coated ^99m^Tc-carbon nanoparticles. **(B)** Representative SPECT images of normal rabbit lungs 3 hours after intravenous injection of radiolabeled microspheres of different sizes: 30 µm (a), 12 µm (b), and 8 µm (c), and comparison with clinical 99mTc-MAA (d). Microspheres exhibit strong lung retention with decreasing size showing broader distribution, while ^99m^Tc-MAA shows visible kidney uptake indicating leaching. **(C)** Fused SPECT/CT images (coronal, sagittal, and transaxial views) of a rabbit liver with a VX2 tumor 1 h post-intra-arterial infusion of 8 µm radiolabeled microspheres, highlighting clear localization and definition of the tumor region. Adapted with permission from 113, Copyright © 2019 Taylor & Francis.

^99m^Tc-Nanoparticles composite is emerging as a superior surrogate for ^90^Y TARE, addressing the limitations of conventional ^99m^Tc**-MAA**, which often disaggregates *in vivo*, leading to uneven distribution and potential systemic leakage. 113, 114. The ^99m^Tc-Nanoparticles composite microspheres developed by Stephens *et al.* consists of protamine-coated ^99m^Tc-labeled carbon nanoparticles (FibrinLite; 150–350 nm), electrostatically attached to polystyrene sulfonate microspheres (30 µm, 12 µm, and 8 µm), using the same polymer base as clinically used ^90^Y microspheres. 113. The radiolabeling method produces highly stable microspheres that retain ^99m^Tc even after extensive washing and *in vivo* circulation. Scanning electron microscopy has confirmed uniform nanoparticle attachment on the microsphere surface **(Figure 10A)**. In preclinical rabbit models, lung retention of these radiolabeled nanocomposite microspheres ranged from 72.8% (8-µm) to 92.9% (30-µm), compared to only 66.8% for ^99m^Tc**-MAA**, which also exhibited higher systemic leakage (29%) as shown in **Figure 10B**. The retention of the microspheres in normal liver was significantly higher (99.2% to 99.8%), whereas in VX2 tumor-bearing livers, it ranged from 98.2% to 99.2%. The tumor uptake following intrahepatic arterial infusion was 32.0%–33.0% of total liver radioactivity, reflecting increased arterial perfusion due to angiogenesis. Moreover, the smaller microspheres (8 µm) provided detailed visualization and tumor targeting due to their ability to reach finer vasculature **(Figure 10C),** demonstrating their potential for superior dosimetry planning. These findings suggest that radiolabeled nanocomposites are a promising surrogate for therapeutic microspheres, offering improved stability, biodistribution, and imaging precision for more accurate pre-therapy planning in clinical TARE of HCC. However, further preclinical validation, long-term biocompatibility assessments, and clinical trials are needed before widespread clinical adoption.

## 5. Pre-treatment Planning and Dosimetry of TARE

The basic concept behind TARE therapy is the dual blood supply in liver, where tumor receives blood predominantly from hepatic artery, while normal liver parenchyma is mainly supplied from portal vein, enabling intra-arterial delivery of radioactive microspheres 11. This technique facilitates selective and preferential accumulation of microspheres within tumor microvasculature, resulting in highly localized radiation exposure to tumors. The important component of TARE includes dosimetry planning, allowing the estimation of patient-specific absorbed dose that ensures maximum radiation dose to tumors while reducing the exposure to normal liver tissues. The prior studies reported a threshold dose of ~100 Gy was considered sufficient for controlling tumor, while exposure < 30 Gy to normal liver prevent radiation-induced liver disease (RILD) 115, 116. However, the results from the LEGACY study and the recent clinical trials have proposed the threshold doses according to the treatment methods (segmental vs. lobar) and microsphere types (glass vs. resin) 117. These studies demonstrate that in radiation segmentectomy, ablative doses often exceed 400 Gy with glass microspheres, while 190-200 Gy is typically recommended for resin microspheres. In contrast, for lobar treatment, the DOSISPHERE-01 trial using glass microspheres showed that glass microspheres can deliver up to 250 Gy to the tumor dose while sparing healthy liver tissue, whereas the tumoricidal dose for resin microspheres is lower typically between **100 Gy and 120 Gy** due to the differences in particle characteristics and dosimetry behavior 118. Dosimetry methodologies in TARE are categorized as pre-treatment (planning) or post-treatment (verification) approaches.

The TARE workflow begins with baseline imaging (contrast-enhanced CT, MRI, or PET/CT) to assess tumor burden followed by mesenteric angiography to precisely map the hepatic arterial anatomy and identify any extrahepatic vessels 11. Subsequently, intra-arterial administration of surrogate microspheres (predominantly ^99m^Tc-MAA) is performed using angiographic guidance, and planar scintigraphy or SPECT imaging to assess microspheres distribution and enable pre-treatment dosimetry 119. Pre-treatment imaging and dosimetry provide prior knowledge of absorbed dose and lung shunt fraction estimates for patient-specific tumor targeting during TARE therapy. The threshold dose for normal liver parenchyma is ~ 60 Gy for glass microspheres however, it is ~ 40 Gy only for resin microspheres due to their higher particle number and lower specific activity. 120. An excessive lung shunt fraction increases the risk of radiation-induced pneumonitis, especially when the lung dose >30 Gy in a single treatment or 50 Gy cumulatively 121. Post-treatment bremsstrahlung SPECT (bSPECT) or ^90^Y PET imaging is generally after the administration of therapeutic radioactive microspheres to evaluate post-treatment dose distribution and treatment response. This section summarizes the strengths and limitations of conventional pre-treatment dosimetry methods and highlights the recent advances in patient-specific absorbed dose calculation approaches.

### 5.1. Body Surface Area (BSA) Method

BSA method is a simple and semi-empirical dosimetry approach widely used for resin microspheres, estimates administered activity based on the body surface area of a patient 122, 123. This method does not require ^99m^Tc-MAA SPECT/CT imaging for treatment planning, making it computationally fast and valuable in clinical settings 122. However, this method has several limitations such as it does not account for the variations in tumor-to-normal liver (T/N) ratio that potentially results in suboptimal dose distribution. Because this method assumes a fixed relationship between patient size and liver volume, it may lead to overestimation or underestimation of required activity, particularly in large patients with smaller livers, and vice versa 114.

### 5.2. Single-Compartment Medical Internal Radiation Dose (MIRD) Model

The single-Compartment MIRD model is considered as standard dosimetry method in clinical TARE therapy, particularly when using glass microspheres. However, this MIRD model is based on assumptions that the activity is uniformly distributed in source regions and does not consider macroscopic and microscopic uptake and absorbed dose heterogeneity 122, 124. The targeted liver is treated as a single homogenous compartment to estimate required activity of therapeutic microspheres. As mentioned above, this method does not account for spatial heterogeneity in microsphere distribution, limits the dosimetry accuracy in patients with variable T/N uptake and may lead to underdosing of tumor or overdosing of normal liver parenchyma 114. These drawbacks have limited the widespread adoption of this model in clinical settings.

### 5.3. Partition Model (PM) and Multi-tumor Partition Model (MTPM)

In the Partition model, the liver is divided into multiple compartments (tumor, normal liver, and lung compartments). Because the dosimetry is still based on MIRD schema, it is also commonly referred as multi-compartment MIRD model. Compared to the BSA and single-compartment MIRD models, the PM is more accurate in activity estimation and applicable to both glass and resin microspheres. This method integrates ^99m^Tc-MAA SPECT/CT imaging in treatment planning to incorporate T/N ratios, it provides more accurate and personalized activity estimation, maximizing tumor-absorbed dose (≥205 Gy for glass microspheres) while minimizing radiation exposure to normal liver and lungs 125, 126. The results from DOSISPHERE-01 trial highlight the benefits of PM when using ^99m^Tc-MAA SPECT/CT. It significantly improved objective response rates (71% vs. 36%, p=0.0074) and overall survival (26.6 months vs. 10.7 months, p=0.0096) in HCC patients compared to standard dosimetry. PM-based personalized dosimetry achieved 96% objective response rate (tumor-absorbed doses ≥205 Gy) compared to only 42% in the standard dosimetry group (tumor-absorbed dose ~120 Gy), confirming that higher tumor doses significantly correlate with improved survival and response rates without increasing treatment-related toxicity 118. Although PM is more accurate and safer, its application remains limited due to the requirement of high-resolution imaging and time-consuming volume determination steps for precise uptake measurements 11. Furthermore, PM assumes uniform dose distribution within each compartment; it may not be accurate for highly heterogeneous tumors, leading to suboptimal therapy 119.

Another dosimetry approach called MTPM was formulated to mitigate the above limitations. In MTPM, each tumor is treated as an independent compartment with its own T/N ratio and absorbed dose estimations, accounting for inter-tumor variability in vascularity and microsphere uptake to enhance dose accuracy 114, 125. This model performs differential dosing in each lesion, ensuring effective radiation dose without non-specific radiation. MTPM integrates effectively with ^99m^Tc-MAA SPECT/CT image-based voxel dosimetry and provides 3D dose distribution maps 125. Comparative studies between PM and MTPM demonstrated closer alignment of MTPM with advanced 3D voxel-based dosimetry models that is confirming its superior accuracy in dose prediction. When 3D voxel dosimetry is not available, MTPM represents the optimal alternative for personalized dosimetry.

### 5.4. Image-based 3D-Voxel Dosimetry in TARE

The Local Deposition Model (LDM) and Dose Point Kernel (DPK) convolution Model are the representative voxel-based methods that account for spatial activity distribution providing voxel-level absorbed dose estimation using SPECT/CT imaging data. LDM is assuming that all beta energy from ^90^Y-microspheres is deposited within the voxel containing the radioactivity. This method enables computationally efficient dose calculations by converting ^99m^Tc-MAA SPECT counts into ^90^Y activity concentrations with assuming entrapped microsphere in liver tissue. Moran *et al.* showed that LDM yielded mean tumor absorbed doses 20–30% higher than PM predictions, particularly in patients with multiple tumors 125. Since LDM does not consider beta particle cross-absorbed dose between adjacent voxels, it underestimates dose in highly perfused regions, limiting its application 114. The DPK convolution model convolves a precomputed dose point kernel with the cumulative activity distribution from ^99m^Tc-MAA SPECT to determine absorbed dose. In contrast to LDM, the DPK method also incorporates beta-particle cross-absorbed dose between the neighboring voxels, estimating more accurate absorbed dose as demonstrated by Moran *et al.*, where DPK provided ~ 22% higher tumor absorbed compared to PM 125. Their study also found DPK dosimetry correlated more closely with clinical outcomes than LDM, particularly for tumors with heterogeneous microsphere distribution. Similarly, Kim* et al.* reported that DPK convolution significantly improved dose estimation for large tumor volumes where cross-irradiation effects are more pronounced 119.

### 5.5. Advances in Pre-Treatment Dosimetry Using Surrogate Imaging Agents

**Figure 11:**
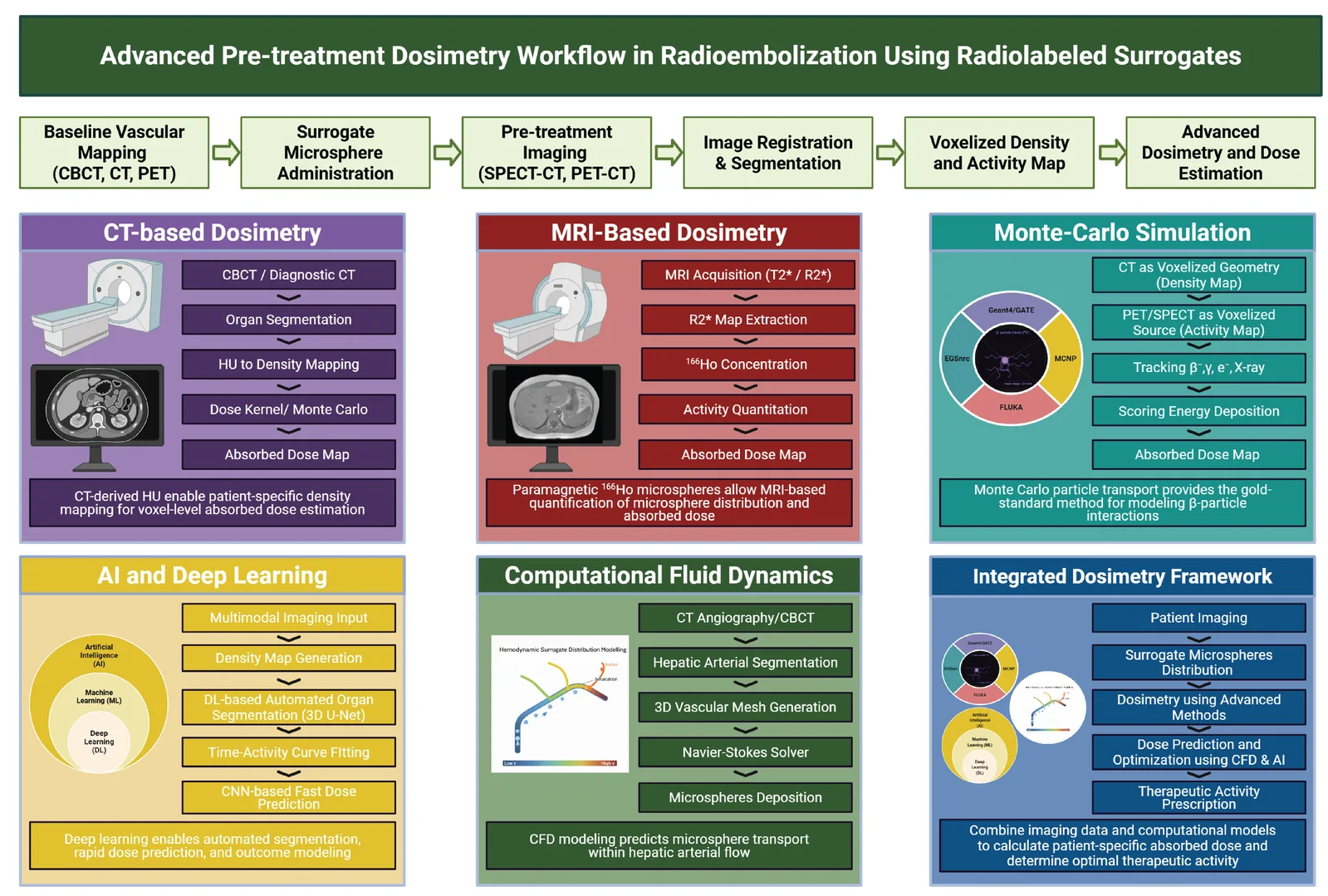
Advanced pre-treatment dosimetry workflow in TARE using radiolabeled surrogate microspheres. An integrated framework for patient-specific dosimetry including sequential baseline vascular mapping, surrogate microsphere administration, and pre-treatment imaging, followed by image registration and voxelized density-activity mapping. MRI/CT-based dosimetry, MC radiation transport simulation, AI–assisted analysis, and computational fluid dynamics modeling of microsphere are combined to predict microsphere distribution and absorbed dose, enabling optimized activity prescription and personalized TARE planning.

The recent advancement in medical imaging infrastructures and image reconstruction algorithms have facilitated the way for more accurate pre-treatment imaging and dosimetry. Moreover, the accuracy of surrogate-based pre-treatment dosimetry approaches has improved, transitioning from compartmental models to high resolution voxel-based and multimodal techniques **(Figure 11).** These methodological advancements enable better mapping of heterogenous microsphere distribution and estimation of patient-specific absorbed dose.

#### 5.5.1. CT-based dosimetry

**Figure 12:**
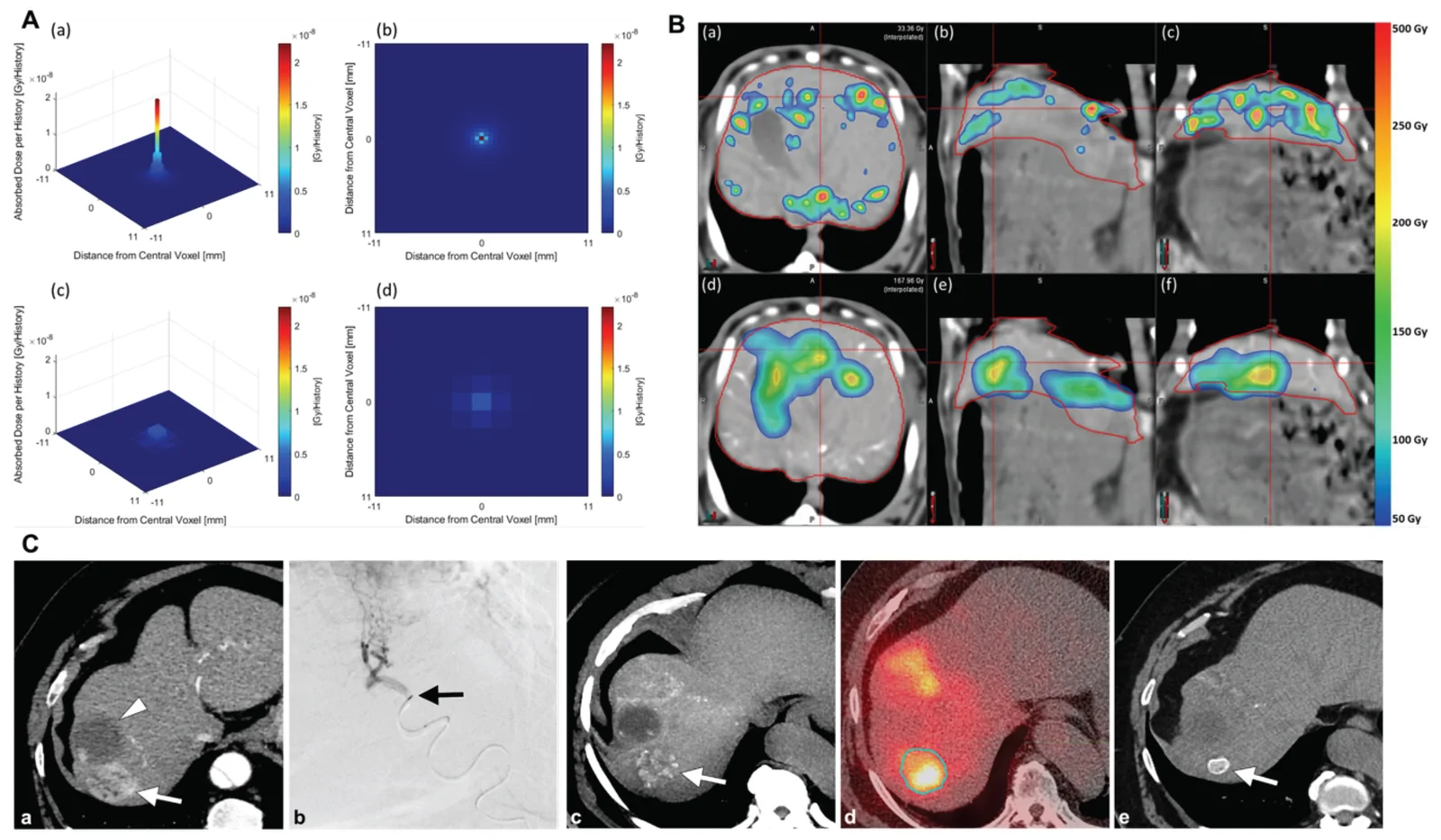
CT-based dosimetry. **(A)** MC-derived dose-voxel kernels (DVKs) for ^90^Y. CT-specific DVK with higher spatial resolution shows concentrated dose in the central voxel (a, b). PET-specific DVK with coarser voxel size demonstrates broader, more diffuse dose spread (c, d). Both DVKs are normalized per history in a water-equivalent voxel matrix. **(B)** Comparison of CT- and PET-based dose distributions in a rabbit liver following administration of radiopaque Eye90 microspheres. CT-based dose distribution (DD_CT_) overlaid on CT slices, revealing highly heterogeneous and localized hotspots aligned with embolized vasculature (a–c). PET-based dose distribution (DD_PET_) shows smoothed, lower-resolution dose maps with fewer identifiable hotspots due to partial volume effects and respiratory motion (d–f). Adapted with permission from 103, Copyright © 2022 Springer Nature. **(C)** Multimodal clinical imaging of ^90^Y-radioembolization with radiopaque microspheres. Pre-treatment contrast-enhanced CT shows a 4.3-cm tumor in segment 8 (arrowheads; a). Digital subtraction angiography confirming catheter placement (black arrow; b). Post-treatment non-contrast CT confirms radiopaque Eye90 microsphere distribution (white arrow; c). Post-treatment Bremsstrahlung SPECT/CT demonstrates Eye90 microsphere radioactivity within the tumor (outlined by the circular contour) and the treated volume, closely matching the microsphere radiopacity distribution observed on CT in figure 2c (d). Non-contrast CT in follow-up confirms persistent radiopacity in embolized vessels (white arrow; e), supporting potential role as a long-term treatment biomarker (e). Adapted with permission from 8, Copyright © 2024 Elsevier.

Leveraging the high spatial resolution of CT, post-treatment imaging using radiopaque microspheres is a promising method for assessing microsphere distribution and estimating absorbed dose in ^90^Y radioembolization 8, 102, 103. In contrast to PET and SPECT imaging, which are limited by lower spatial resolution and motion artefacts, CT imaging offers greater detail for evaluating dose heterogeneity. Preclinical studies using imageable microspheres, such as Eye90, have demonstrated strong correlation between microsphere distribution and absorbed dose, validating its potential for post-treatment dose confirmation. For example, in a porcine renal model, micro-CT based activity mapping yielded mean dose within 5.7% of the MC-calculated reference values. Dose-volume metrics such as D70 (the dose delivered to 70% of the target volume) values were shown to strongly correlate with voxel size (R² = 0.90), further underscores the advantage of high-resolution CT imaging for accurate dose distribution 102. Similarly, a rabbit liver study, utilizing a CT calibration phantom showed strong correlation (R² > 0.999) between Hounsfield units and microsphere concentration, enabling accurate voxelized dose assessment as shown in **Figure 12A-B** 103. The mean dose in CT-derived distributions showed greater heterogeneity with a coefficient of variation of 1.99, compared to 1.02 in PET, which highlights the improved precision of CT imaging. Recent clinical studies have continued to validate this dosimetry approach, showing that radiopaque microspheres not only enhance visualization but also improve tumor targeting accuracy when combined with SPECT imaging 8. In a clinical trial, Abraham *et al.* demonstrated precise CT-based visualization of microsphere distribution, which correlated with SPECT-based dosimetry. In their study, the administered activity ranged from 0.4 GBq to 9.6 GBq, achieving tumor doses exceeding 250 Gy in some cases, while keeping doses to normal liver tissues safe. **Figure 12C** demonstrates strong visual correlation between microsphere radiopacity on post-treatment CT and radioactivity on SPECT/CT, confirming accurate tumor targeting. Overall, post-treatment CT imaging provides an effective means to improve spatial resolution, mitigate respiratory motion artifacts, and provide a comprehensive assessment of dose heterogeneity, thereby enhancing visualization of ^90^Y TARE dosing.

#### 5.5.2. MRI-based dosimetry

**Figure 13:**
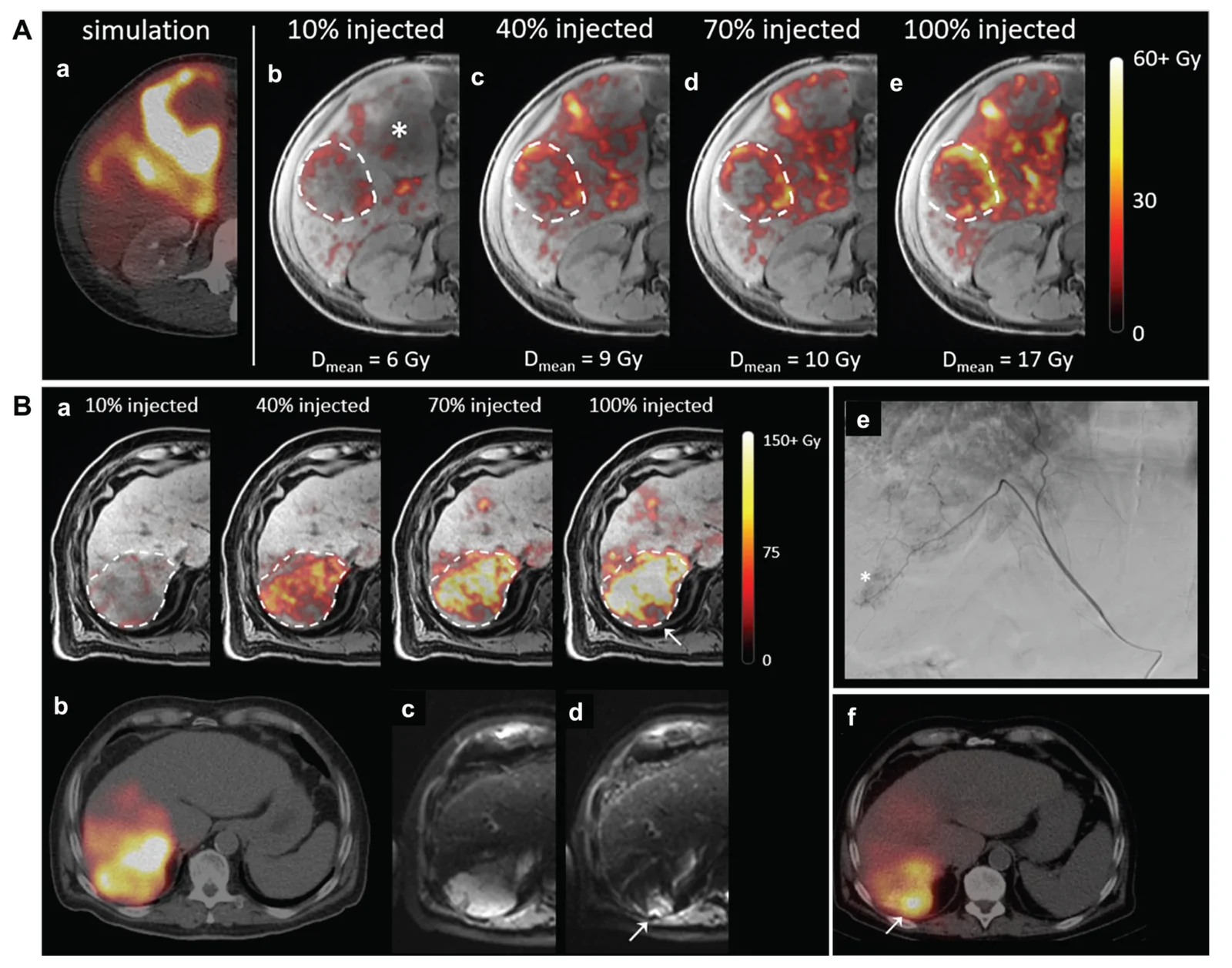
Intraprocedural MRI-based dosimetry during ^166^HoTARE. **(A)** Comparison between pre-treatment ^99m^Tc-SPECT-based dose simulation (a) and intraprocedural MRI-based dose maps acquired after stepwise administration of ^166^Ho-microspheres (10%, 40%, 70%, and 100% activity) in a patient with breast cancer liver metastases (b-e). The mean tumor dose (D*_mean_*) increases from 6 to 17 Gy however, spatial dose distribution remains largely unchanged, suggesting that increased activity alone may not compensate for suboptimal microsphere deposition. **(B)** Another case of HCC showing incomplete tumor coverage due to variant vascular anatomy. MRI-based dose maps after each of four sequential administrations of ^166^Ho-microspheres via the right hepatic artery (a), a portion of the tumor (dashed line) remained undertreated due to vascularization from an aberrant phrenic artery branch (arrow). Pre-treatment ^99m^Tc-SPECT/CT simulation showing predicted microsphere distribution (b). Diffusion-weighted MRI before (c) and 3 months after (d) treatment shows persistent diffusion restriction in the undertreated tumor region (arrow). Digital subtraction angiography visualizing the aberrant vessel supplying the residual tumor; the asterisk indicates the persistent tumor vasculature (e). Post-re-treatment ^166^Ho-SPECT/CT after catheterization of the aberrant vessel, showing high microsphere uptake in the previously undertreated area (arrow), indicating successful targeting during the second intervention (f). Adapted with permission from 127, Copyright © 2022 Springer Nature.

MRI-based dosimetry has evolved from a theoretical concept into a clinically viable tool for precise radiation dose estimation in TARE, leveraging the paramagnetic properties of ^166^Ho- microspheres to enable direct imaging with MRI 15, 29, 127. The recent clinical trial confirmed the feasibility and potential superiority of MRI-based dosimetry compared to SPECT-based methods 127. Seevinck *et al.* first demonstrated MRI-based quantification of ^166^Ho-PLLA-MS using a DPK convolution method, showing a high correlation (R² = 0.99) with SPECT-based dosimetry 29. MRI-based dosimetry involves pre-treatment T2-weighted imaging, voxel-wise transverse relaxation quantification for microsphere concentration, and MC modeling for dose map generation 29. Recently, Roosen *et al.* introduced intraprocedural MRI-based dosimetry in the EMERITUS-1 clinical trial, enabling dynamic treatment adjustments to optimize tumor coverage, minimize toxicity, and improve administered activity detection by an average of 5.8% 127. Their study reported a case where ^99m^Tc-MAA SPECT/CT inaccurately showed complete tumor coverage that results in undertreatment of a portion of tumor supplied by an aberrant phrenic artery. MRI-based dosimetry however, identified the underdosed portion precisely and facilitated retreatment using ^166^Ho TARE, emphasizing the critical advantage of real-time MRI-based dosimetry in identifying perfusion mismatches and optimizing treatment strategies **(Figure 13)**. Because of higher spatial resolution of MRI compared to SPECT, MRI-based dosimetry demonstrated reduced dosimetry uncertainties (5–10% errors), outperforming SPECT-based uncertainties (10–20% errors). MRI-based dosimetry has several advantages including non-ionizing imaging for microspheres localization however, its clinical utilization remains limited due to susceptibility and motion artifacts of MRI and high costs which are highly relevant to emerging MRI-visible agents. Recent advances and future developments in MRI infrastructures and such as hybrid PET/MRI imaging, MRI-compatible injection systems, artefacts-free MR imaging, AI-driven dose calculations, and large-scale clinical trials, could help establish its role in next-generation image-guide TARE.

#### 5.5.3. Monte Carlo Simulation

**Figure 14:**
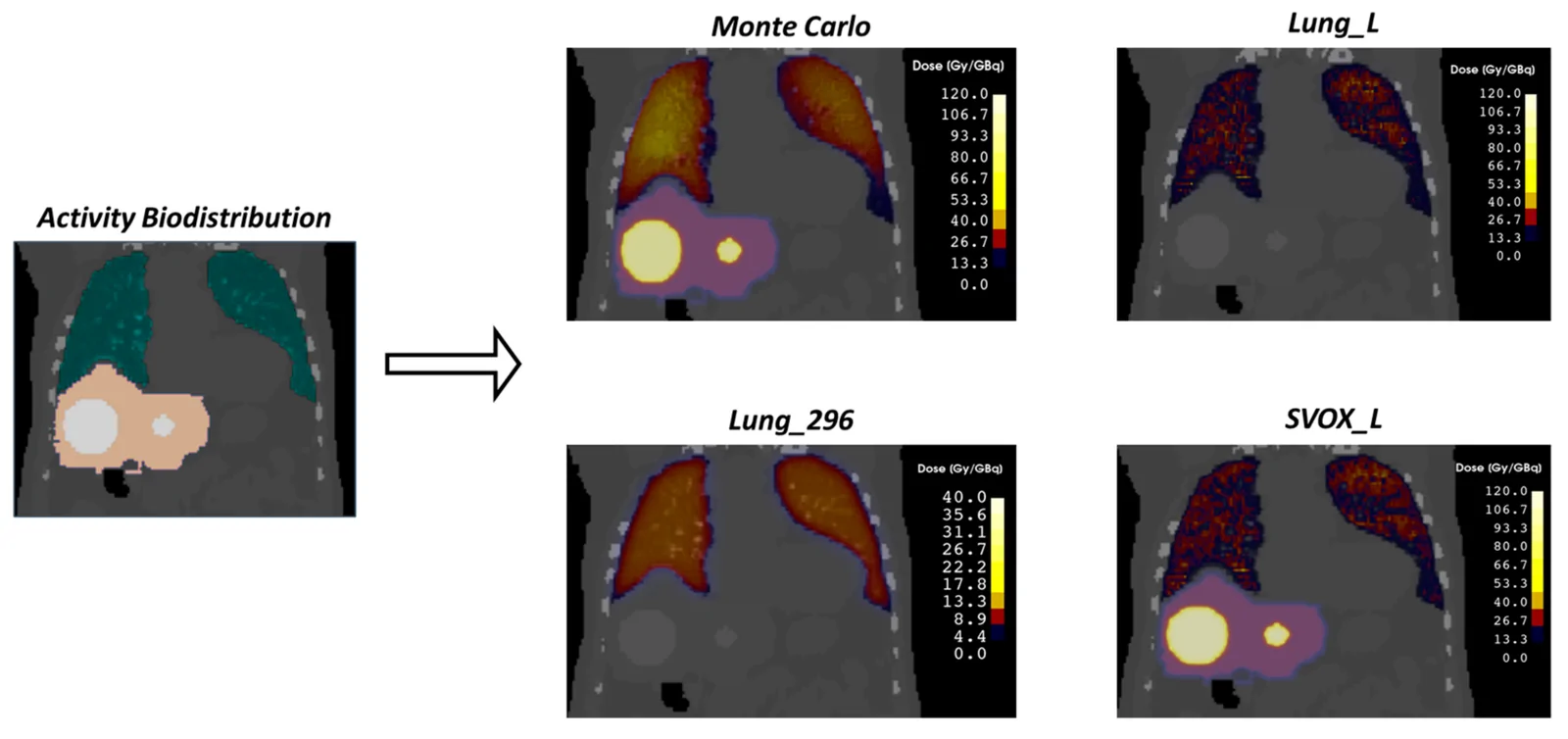
Comparison of absorbed dose distributions obtained from MC simulations and classical voxel-based methods for a lung shunt (LS) of 20%. From left to right, this figure shows activity biodistribution, followed by the AD distribution maps from MC simulations and several convolution-based approaches (SVOX_L, Lung_296, and Lung_L) for visual comparison. The colors used to depict activity biodistribution are illustrative, indicating a uniform distribution within each region. For Lung_296 and Lung_L, AD distributions exclude the liver region due to the computational choice to crop the activity map to the lungs. AD values are reported in Gy per GBq of administered activity, with Lung_296 represented on a different color scale (0 to 40 Gy/GBq) compared to other methods (0 to 120 Gy/GBq) to ensure visibility of dose values. Adapted with permission from 131, Copyright © 2024 MDPI.

MC simulation is widely regarded as gold standard in voxel-based dosimetry by explicitly modeling radiation transport and patient-specific tissue heterogeneity using CT-derived phantoms and SPECT-based activity distributions, providing highly accurate absorbed dose estimation in RPT and TARE therapy 128-130. Despite its advantages in integrating anatomical and functional heterogeneity, studies comparing MC simulation to image-based methods have yielded mixed results, with some showing no significant differences in dose calculations 114. However, a prior study reported that MC-derived doses were, on average, 25–40% higher than those obtained from standard PM method, reflecting the latter’s tendency to underestimate dose deposition in non-uniform activity regions 125. d’Andrea *et al.* highlighted the role of MC simulations for lung dosimetry in ^90^Y TARE that resulted in ~ 88% improved accuracy in absorbed dose estimation at voxel-level compared to conventional dosimetry methods, underscoring its reliability in reducing uncertainties in lung shunt estimation and improving treatment efficacy **(Figure 14)** 131. MC dosimetry method remains computationally intensive and time-consuming, limiting its clinical application. The ongoing efforts to develop GPU-accelerated MC algorithms aim to overcome these limitations.

#### 5.5.4. Artificial Intelligence and Deep Learning

AI and DL have been extensively used in nuclear medicine imaging and RPT dosimetry to enhance accuracy, efficiency, and clinical scalability. MC-based dosimetry method provides highly accurate dose estimates at voxel-level but remains computationally intensive and time-consuming, limiting their routine clinical application. To address this issue, recent studies have demonstrated that AI- and DL-based dosimetry models can approximate MC-level accuracy in dose estimates with substantially reduced computation time 132-134. Recent studies have demonstrated that AI models can approximate MC-level dose calculations with substantially reduced computation time. Kim *et al.* develop a DL-based dosimetry framework that estimate dose comparable to MC simulations while significantly reducing computational burden 135. Ha *et al.* reported that the DL method capable of predicting voxel-level dose distributions with strong agreement to MC simulations (R2 up to 0.99), while reducing computation time from approximately 4.6 hours to 2.6 minutes 136. Importantly, their model demonstrated the ability to generalize across radionuclides without radionuclides-specific training data, addressing a major limitation of prior AI-based dosimetry approaches. Furthermore, Ahn *et al.* showed that DL model can accurately predict patient-specific dose distribution directly from anatomical inputs, outperforming conventional knowledge-based planning approaches significantly reducing planning time 137. In the context of ^90^Y-TARE, these capabilities are particularly relevant. Plachouris *et al.* demonstrated that DL models can predict post-treatment ^90^Y microspheres distribution from pre-treatment ^99m^Tc-MAA SPECT/CT. Mean absorbed dose differences of 5.42% ± 19.31% in tumors and 0.44% ± 1.64% in the liver were identified 138. The results of this study revealed improved predictive performance when compared to conventional methods, which often fail to consider the differences in particle characteristics and therapeutic flow dynamics. In addition, dosimetry frameworks based on AI enable accelerated dose calculations at voxel-level, reducing dependencies on time-consuming MC simulations while preserving spatial accuracy. AI and DL-based auto segmentation of normal liver and liver tumors reduce inter- and intra-operator variability and increase reproducibility in absorbed dose estimations. In contrast to multi-timepoints imaging-based dosimetry, AI-based models capable of estimating activity distributions from limited imaging data, improving clinical workflow and patient comfort. Furthermore, AI can integrate imaging, clinical, and biological data to improve accuracy in dose calculation, identify toxicity risk factors, and enable adaptive treatment planning 139. In a recent study, Woo *et al.* highlighted that DL approaches, including U-Net and generative adversarial networks (GANs) improve image quality, auto segmentation, and accuracy in voxel-level dose estimation while enhancing efficiency and reproducibility in dosimetry 140. Despite this promising development, the clinical integration of AI in ^90^Y TARE remains at an early stage. Most of the available models are trained on limited datasets and lack generalizability across different imaging systems. Moreover, AI-based approaches typically rely on MC or voxel-based dosimetry as reference standards, indicating that further validation, standardization, and multi-center studies are required before widespread clinical adoption.

#### 5.5.5. Advanced Multimodal Imaging

Recent advancements in medical imaging infrastructures, image registration tools and image reconstruction algorithms have emphasized a multimodal imaging approach that integrates cone-beam CT, angio-CT, and ^18^F-Fluoro-deoxyglucose (^18^F-FDG) PET to generate a comprehensive patient-specific liver map 141, 142. These advanced imaging methods exploit the unique strengths of each modality to improve tumor visualization, vascular anatomy assessment, and dosimetry precision for optimized treatment planning. Furthermore, PET-imaging based dosimetry using ^86^Y is emerging in ^90^Y-TARE as a superior alternative to ^99m^Tc-MAA SPECT/CT for pre-treatment planning, as it more accurately predicts the biodistribution of therapeutic microspheres and reduces discrepancies between planned and actual dose 143. In addition, Cherenkov luminescence imaging (CLI) has been gaining importance in ^90^Y-TARE application. CLI is an optical imaging modality that detects visible photons emitted from ^90^Y, offering a label-free strategy to track ^90^Y-microspheres in TARE without exogenous modifications or reporters. As reviewed by Teng *et al*., CLI allows real-time visualization of tumor margin, with direct applicability to monitor intraprocedural ^90^Y microspheres distribution and post-treatment dosimetry 144. The high energy pure β-emission of ^90^Y is beneficial to CLI sensitivity while eliminating γ-radiation background. This positions CLI as a promising non-invasive complement to ^90^Y-PET/CT in precision TARE management. Moreover, incorporating radiomics and genomic data into dosimetry models will permit more precise predictions of tumor response and toxicity, paving the way for truly personalized treatment strategies 145.

#### 5.5.6. Computational Fluid Dynamics

Another significant advancement in TARE is the application of computational fluid dynamics (CFD) modeling. CFD-based dosimetry software simulates the intrahepatic distribution of ^90^Y microspheres, enabling precise predictions of therapeutic agent dispersion within the hepatic vasculature 146, 147. By incorporating vessel morphology/structure, arterial flow velocity profiles, and microsphere physical properties, CFD-based dosimetry frameworks may transcend the inherent limitations of population-averaged partition models and empirical BSA-based activity prescriptions, enabling truly individualized pre-treatment simulation of ^90^Y microsphere dispersion within the tumor-bearing hepatic vasculature. A dedicated CFD modeling study reported discrepancies of 2% to 3.5% between ^90^Y distributions predicted using ^99m^Tc-MAA and actual in vivo distributions assessed via ^90^Y PET/CT 147. This is underscoring the superior predictive fidelity of CFD simulation as a complementary or surrogate-independent planning tool. As clinical adoption increases and technical challenges are addressed, these advanced personalized dosimetry strategies can play a crucial role in optimizing ^90^Y TARE outcomes, ultimately leading to more effective and safer treatments for patients with liver cancer.

## 6. Practical Challenges and Considerations in Clinical Translation

Despite their promising attributes, novel surrogate materials face substantial logistical, regulatory, and economic barriers that must be systematically addressed before widespread clinical adoption in TARE can be achieved. At the workflow level, adoption of new surrogate materials requires significant modifications of radiopharmacy procedures, imaging protocol adjustment and structured clinician training to account for surrogate-specific pharmacokinetics and biodistribution patterns. Consistency between pre-treatment simulations and actual ⁹⁰Y microsphere distributions should be thoroughly validated for each candidate surrogate, as intravascular flow dynamics and embolic properties may differ meaningfully between surrogate and therapeutic particles 114. The regulatory pathways present an independent barrier. Clinical integration of new surrogate materials requires rigorous validation of safety and efficacy through extensive preclinical and clinical trial programs establishing equivalence or superiority over existing standards. FDA or EMA approval further mandates adherence to Good Manufacturing Practices (GMP) standard and robust quality control documentation. Infrastructure limitations compound these challenges. Broad deployment of PET- or SPECT-compatible surrogates presupposes access to appropriately equipped and optimized nuclear medicine facilities, which remain unevenly distributed across healthcare systems 148. Economic justification, such as a cost-benefit analysis, is a further prerequisite for the clinical implementation of new surrogate materials, particularly in resource-constrained healthcare systems. While surrogate materials with superior dosimetry accuracy have the potential to reduce treatment failure rates and improve long-term patient outcomes, the upfront financial burden associated with new imaging agents, hardware upgrades, and training personnel are substantial 149. Establishing reimbursement policies and cost-effectiveness studies will therefore be pivotal in determining the feasibility of integrating novel surrogate materials into routine clinical TARE practice.

## 7. Future Perspectives and Conclusion

Over the years, development of surrogate materials for pre-treatment planning of TARE has become central to bridge the gap between pre-treatment imaging and actual therapeutic radioactive microsphere deposition for enhancing therapeutic outcomes. 150. Several emerging surrogate materials have shown meaning advances over conventional surrogates for refining pre-treatment dosimetry. PET-compatible biodegradable microspheres are suggested as next generation alternatives, offering improved dosimetry fidelity while mitigating the long-term embolic risks associated with permanent microspheres. Theranostic radionuclides labeled microspheres further integrated imaging and therapeutic into a single platform, allowing real-time dosimetry assessment and supporting personalized treatment strategies. Multimodal PET/CT/X-ray/MRI visible surrogate materials along with scout-dose strategies have demonstrated potential for improved spatial mapping of deposition and dose estimation. At the same time, MRI or CT visible nanoparticles-based systems including iron oxide and radio-opaque metal nanoparticles have been under active investigation as adjuvants for enhancing dosimetry prediction accuracy 151, 152. In parallel, computational and AI-driven approaches are evolving in concert with these material advances. MC simulations, DL-based biodistribution prediction models, and computational fluid dynamics modeling now provide mechanistic frameworks for anticipating surrogate materials and microspheres distribution across patient specific vascular structures. AI algorithms incorporating patient-specific anatomical and physiological factors increasingly refine personalized dose planning at a level of granularity not achievable through conventional approaches 114. In summary, these converging advances in surrogate materials, dosimetry modeling, and predictive computation approaches are anticipated to lead to improved tumor response rates, extended progression-free survival, and a reduction in complications associated with off-target radiation. Future research should prioritize optimizing and validating these technologies and focus on facilitating their integration into clinical TARE workflows to fully realize their potential in improving patient outcomes.

## Figures and Tables

**Table 1:** Radionuclides for TARE

Radionuclides	Half-life	Emission Type	β⁻ Energy (MeV) Max. (Mean)	γ Energy (KeV)	β⁻ Range in Tissue (mm) Max. (Mean)	Imaging Modalities	Application
Phosphorus-32 (^32^P)	14.29 d	β⁻ (100%)	1.71 (0.695)	No γ emission	7.9 (3)	Bremsstrahlung SPECT	Therapy only
Yttrium-90 (^90^Y)	64.1 h	β⁻ (100%)	2.28 (0.933)	No γ emission	11 (2.5)	Bremsstrahlung SPECT/^90^Y PET	Therapy only
Iodine131 (^131^I)	8.04	β⁻ (86%), γ (81%)	0.606 (0.192)	364	2.5 (0.8)	SPECT	Theranostics
Praseodymium-142 (^142^Pr)	19.12 h	β⁻ (86%), γ (3.7%)	2.16 (0.809)	1.57	8.8 (3)	SPECT	Theranostics
Samarium-153 (^153^Sm)	46.3 h	β⁻ (20%), γ (28%)	0.81 (0.233)	103	3 (0.8)	SPECT	Theranostics
Holomium-166 (^166^Ho)	26.8 h	β⁻ (50.5%), γ (6.7%)	1.84 (0.665)	81	8.7 (3)	SPECT/MRI	Theranostics
Ytterbium-175 (^175^Yb)	4.16 d	β⁻ (86.5%), γ (1.9%)	470 (0.150)	133	2 (0.6)	SPECT	Theranostics
Lutetium -177 (^177^Lu)	6.7 d	β⁻ (78.6%), γ (11%)	497 (0.134)	208	2.2 (0.6)	SPECT	Theranostics
Rhenium-186 (^186^Re)	3.72 d	β⁻ (70%), γ (9.4%)	1.07 (0.323)	137.2	4.5 (1.5)	SPECT	Theranostics
Rhenium-188 (^186^Re)	16.9 h	β⁻ (71.6%), γ (15%)	2.22 (0.765)	155	11 (3.5)	SPECT	Theranostics

**Table 2:** Commercially available Clinical Therapeutic Microspheres for TARE

Properties	Type of Radioactive Microspheres/Particles		
	TheraSphere	Sir-Spheres	QuiremSpheres
Radionuclide	^90^Y	^90^Y	^166^Ho
Half-life (h)	64.1	64.1	26.8
E_β⁻_ (MeV)	2.28	2.28	1.84
E_γ_ (KeV)	No γ emission	No γ emission	80.6 (6.7%)
Matrix Material	Glass	Resin	PLLA
Diameter (µm) - Range (Mean)	20-30 (25)	20-60 (32)	15-60 (30)
Density (gcm^-3^)	3.3	1.6	1.4
Specific Activity (Bq/microsphere)	2500	50	450
Imaging Type	Bremsstrahlung SPECT; ^90^Y PET	Bremsstrahlung SPECT;^90^Y-PET	SPECT; MRI
Application	Therapy	Therapy	Theranostics

**Table 3:** Theranostic Radioactive Microspheres loaded with Biodegradable and Non-degradable Materials

Radionuclide	Microspheres/Particles Materials	Degradability	Particles Size (µm)	Infusion Route	Application	Pre-treatment Imaging	References
^166^Ho	Alginate	Biodegradable	159 ± 19	Intra-arterial	Pre-clinical TARE in Liver Cancers	MRI, SPECT	28
	PLLA	Biodegradable	30 ± 5	Intra-arterial	Pre-clinical and Clinical TARE in Liver Cancer	MRI, SPECT	14, 15, 29-38
	PLLA (QuiremScout™)	Biodegradable	30 ± 5	Intra-arterial	Clinical Scout Dose/ Treatment Planning	MRI, SPECT	36
	Biorex™ 70 resin	Non-degradable	30–100	Intra-arterial	Pre-clinical TARE	MRI, SPECT	31
		
^188^Re	HSA, PLA, collagen, PMMA	Biodegradable & non-degradable	10–150	Intra-arterial	Clinical TARE in HCC	SPECT	39, 40
	Starch-based microparticles	Biodegradable	10–100	Intra-arterial	Preclinical TARE in HCC	SPECT	41
	Lipiodol	Non-degradable	NA	Intra-arterial	Clinical TARE in HCC	SPECT	42, 43
	PLLA microspheres	Biodegradable	13–48	Intravenous/ Intra-arterial	Preclinical TARE in HCC	SPECT	44, 45
		
^153^Sm	Amberlite IR-120 resin	Non-degradable	20–40	Intra-arterial	Preclinical TARE in HCC	SPECT	46
	Acrylic microspheres	Non-degradable	35	Intra-arterial	Preclinical TARE in HCC	SPECT	47
	PLLA microspheres	Biodegradable	~35	Intra-arterial	Preclinical TARE in HCC	SPECT	48
	Polystyrene microspheres	Non-degradable	~33	Intra-arterial	Preclinical TARE in HCC	SPECT	49
	PHBV microspheres	Non-degradable	~30	Intra-arterial	Preclinical TARE in HCC	SPECT	50
	Polymethacrylate microspheres	Non-degradable	~29	Intra-arterial	Preclinical TARE in HCC	SPECT	51
		
^131^I	Gelatin Microspheres	Biodegradable	50-70	Intratumoral	Local Radionuclide Therapy in HCC	SPECT	52 53
	Lipiodol	Non-degradable	NA	Intra-arterial	Clinical TARE in HCC	SPECT	54
	Chitosan Hydrogels	Biodegradable	100–120	Intra-arterial	Preclinical TARE in HCC	SPECT	55
	Chitosan-Collagen Composite	Biodegradable	10–15	Intra-arterial	Preclinical TARE in HCC	SPECT	56
	Silk Fibroin Microspheres	Biodegradable	11	Intra-arterial	Preclinical TARE in HCC	SPECT	57
		
^177^Lu	Chitosan	Biodegradable	36.5±5.3	Intra-arterial	Preclinical TARE in HCC	SPECT	58
	Polydopamine + silica	Non-degradable	~25	Intra-arterial	Preclinical TARE in HCC	SPECT	59
	Alginate	Biodegradable	2-40	Intra-arterial	Preclinical TARE in HCC	SPECT	60
	PLGA	Biodegradable	25	Intra-arterial	Preclinical TARE in HCC	SPECT	61
	Chitosan + Polydopamine + MgO	Biodegradable	20–30	Intra-arterial	Preclinical TARE in HCC	SPECT	62
		
^175^Yb	PLLA	Biodegradable	20–40	Intra-arterial	Preclinical TARE in HCC	SPECT	63
		
^142^Pr	Rare-earth aluminosilicate glass	Non-degradable	Not confirmed	Intra-arterial	Brachytherapy for hepatic tumors	SPECT	64

**Table 4:** SPECT vs. PET Imaging Characteristics

Imaging Modalities	Characteristics			
	Type of Radiation Used	Imaging Probe	Sensitivity	Spatial Resolution
SPECT	γ-rays	^99m^Tc, ^177^Lu, ^111^In, etc.	10⁻⁹ to 10⁻¹⁰ M (1–10 nM)	6-12 mm (Clinical) 1-2 mm (pre-clinical)
PET	Positron/Annihilation Photons	^18^F, ^86^Y, ^64^Cu, ^89^Zr, etc.	10⁻¹¹ to 10⁻¹² M (10–100 pM)	2-5 mm (Clinical) 1-2 mm (pre-clinical)

**Table 5:** PET-compatible surrogate particles for ^90^Y Microspheres

Radionuclide	Microspheres/ Particles	Degradability	Particles Size (µm)	Administration Route	Application	Imaging Method	Dosimetry Method	References
^89^Zr	Resin	Non-degradable	20-40	Intra-arterial	Pre-clinical	PET	PET Image-based voxel dosimetry & MC simulation	91
^86^Y	Resin	Non-degradable	20-40	Intra-arterial	Pre-clinical	PET	PET Image-based voxel dosimetry & MC simulation	91
^18^F	Resin	Non-degradable	20-40	Intra-arterial	Pre-clinical	PET	PET Image-based voxel dosimetry & MC simulation	92
	Ceramic hydroxyapatite	Non-degradable	20-50	Intra-arterial	Pre-clinical	PET	PET Image-based voxel dosimetry & MC simulation	93
^68^Ga	Starch-based microparticles	Biodegradable	10-100	Intra-arterial	Pre-clinical	PET	PET Image-based voxel dosimetry & MC simulation	41
	Silica	Non-degradable	10-50	Intra-arterial	Pre-clinical	PET	PET Image-based voxel dosimetry & MC simulation	94
	PEI-Alginate	Biodegradable	10-25	Intravenous/ Intramuscular	Pre-clinical	PET	PET Image-based voxel dosimetry & MC simulation	89

## Abbreviations

ADIBO-NHS: azadibenzocyclooctyne N-hydroxysuccinimide ester
AI: artificial intelligence
AVM: arteriovenous malformation
BED: biologically effective dose
CE: conformite europeenne
CT: computed tomography
DL: deep learning
DSA: digital subtraction angiography
DSM: degradable starch microsphere
DVH: dose-volume histogram
FDA: Food and Drug Administration
FTIR: Fourier transform infrared spectroscopy
GMS: gelatin microsphere
HCC: hepatocellular carcinoma
HDE: humanitarian device exemption
HSA: human serum albumin
LLS: liver-lung shunt
MAA: macroaggregated albumin
MC: Monte Carlo
microPet: micro positron emission tomography
MRI: magnetic resonance imaging
MS: microspheres
NOTA: 1,4,7-triazacyclononane-1,4,7-triacetic acid
PBS: phosphate-buffered saline
PDA: polydopamine
PET: positron emission tomography
PHBV: polyhydroxybutyrate-co-3-hydroxyvalerate
PLGA: poly(lactic-co-glycolic acid)
PLLA: poly(L-lactic acid)
PMMA: polymethyl methacrylate
SBMP: starch-based microspheres
SEM: scanning electron microscopy
SFM: silk fibron microspheres
SIRT: selective internal radiation therapy
SPECT: single photon emission computed tomography
TARE: trans-arterial radioembolization
VOI: volume of interest
XRD: X-ray diffraction
